# BMP‐ACVR1 Axis is Critical for Efficacy of PRC2 Inhibitors in B‐Cell Lymphoma

**DOI:** 10.1002/advs.202306499

**Published:** 2024-01-16

**Authors:** Dongdong Liu, Zhen Li, Dongxia Tan, Yang An, Liping Chu, Tiancheng Chen, Weijia Li, Ailin Zhou, Ruijie Xiang, Liye Zhang, Yuxiu Qu, Wei Qi

**Affiliations:** ^1^ Gene Editing Center School of Life Science and Technology ShanghaiTech University 393 Middle Huaxia Road Shanghai 201210 China; ^2^ Shanghai Clinical Research and Trial Center Shanghai 201210 China

**Keywords:** ACVR1, B‐cell lymphoma, biomarker, BMP, cancer therapeutics, PRC2 inhibitors

## Abstract

EZH2 is the catalytic subunit of the histone methyltransferase Polycomb Repressive Complex 2 (PRC2), and its somatic activating mutations drive lymphoma, particularly the germinal center B‐cell type. Although PRC2 inhibitors, such as tazemetostat, have demonstrated anti‐lymphoma activity in patients, the clinical efficacy is not limited to EZH2‐mutant lymphoma. In this study, Activin A Receptor Type 1 (ACVR1), a type I Bone Morphogenetic Protein (BMP) receptor, is identified as critical for the anti‐lymphoma efficacy of PRC2 inhibitors through a whole‐genome CRISPR screen. *BMP6*, *BMP7*, and *ACVR1* are repressed by PRC2‐mediated H3K27me3, and PRC2 inhibition upregulates their expression and signaling in cell and patient‐derived xenograft models. Through BMP‐ACVR1 signaling, PRC2 inhibitors robustly induced cell cycle arrest and B cell lineage differentiation in vivo. Remarkably, blocking ACVR1 signaling using an inhibitor or genetic depletion significantly compromised the in vitro and in vivo efficacy of PRC2 inhibitors. Furthermore, high levels of BMP6 and BMP7, along with ACVR1, are associated with longer survival in lymphoma patients, underscoring the clinical relevance of this study. Altogether, BMP‐ACVR1 exhibits anti‐lymphoma function and represents a critical PRC2‐repressed pathway contributing to the efficacy of PRC2 inhibitors.

## Graphical abstract

Polycomb Repressive Complex 2 (PRC2) inhibitors are lymphoma therapeutics. However, the lack of guiding biomarker limits their clinical application. In this article, authors report that BMP‐ACVR1 signaling reprents a vital PRC2‐repressed pathway contributing to the anti‐lymphoma efficacy of PRC2 inhibitors. Elevated BMP6/7 and ACVR1 correlate with extended survival in lymphoma patients, underscoring its clinical relevance and potential usage as biomarker.

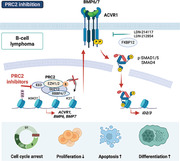

## 1 Introduction

The germinal center (GC) is a structure in secondary lymphoid organs, such as lymphoid nodes, where B cells interact with antigens presented by T follicular helper (Tfh) cell. Within the GC, B cells undergo processes like somatic hypermutation (SHM) and affinity maturation. These processes involve error‐prone mechanisms and may lead to tumorigenesis. In fact, GCs are the primary origin of various B cell non‐Hodgkin lymphomas (NHL), including follicular lymphoma (FL), diffused large B cell lymphoma (DLBCL), and mental cell lymphoma. These lymphomas place a significant burden on healthcare resources. For instance, DLBCL is the most prevalent subtype of NHL, accounting for 31–40% of cases globally, with an estimated incidence of seven cases per 100 000 individuals in US.^[^ ^1^ ^]^

Extensive sequencing efforts and genetic studies in lymphoma research have unveiled new targets and novel therapeutic approaches. Gain‐of‐function (GoF) mutations of *EZH2* have been identified in various B‐cell lymphomas, including FL, DLBCL, and others.^[^ ^2^ ^]^ EZH2 serves as enzymatic subunit of Polycomb Repressive Complex 2 (PRC2), which in turn requires at least SUZ12 and EED subunits for the methylation of histone H3 at lysine 27 (H3K27). The GoF mutations in EZH2 are predominantly located at residue Y641, although also occur at residues W113, A677, and A687. These mutations enhance the trimethylating activity of PRC2, leading to alterations in the epigenomic landscape and transcriptional profiles, and even reorganization of chromatin domains.^[^ ^3^, ^4^, ^5^, ^6^, ^7^ ^]^ Expression of *Ezh2^Y641F^* in B cells or melanocytes has been shown to result in high‐penetrance lymphoma or melanoma in mice,^[^ ^8^, ^9^ ^]^ providing further evidence of the oncogenic nature of these EZH2 GoF mutations.

EZH2 and EED inhibitors have been discovered and demonstrated clinical efficacy in lymphoma patients.^[^ ^10^ ^]^ EZH2 inhibitors directly target the catalytic subunit, while EED inhibitors bind to EED in the H3K27me3 pocket and inhibit PRC2 allosterically. Tazemetostat (EPZ6438), a representative EZH2 inhibitor, was approved by FDA for FL treatment.^[^ ^11^ ^]^ Valemetostat, a EZH1/EZH2 dual inhibitor, was approved for treating adult T‐cell leukemia/lymphoma.^[^ ^12^ ^]^ Several EED inhibitors have also been reported, with MAK683 being studied in clinical trial (NCT02900651).^[^ ^13^, ^14^, ^15^ ^]^ Mechanistically, EZH2's level is closely linked to cell cycle through Rb‐E2F1, and PRC2 inhibitors upregulate cell cycle blocker genes for their anti‐cancer effects.^[^ ^16^ ^]^ PRC2 tri‐methylates H3K27 (H3K27me3), suppressing *p21*/*CDKN1A*, and enabling the proliferation of GC B cells and lymphoma. Additionally, recent research found EZH*2* GoF mutations can attenuate the B cell's requirement for T cell help, drive the expansion of GC centrocytes and initiate FL in mouse model.^[^ ^8^ ^]^ However, the clinical efficacy of tazemetostat and other PRC2 drugs has been observed in FL and DLBCL patients without EZH2 GoF mutations. It is unclear what other factors contribute to the anti‐tumor efficacy of PRC2 inhibitory drugs.

Here, we investigated the mechanism of PRC2 inhibitors in B‐cell lymphoma using a whole‐genome CRISPR/Cas9 knockout (KO) screen and identified the Bone Morphogenetic Protein (BMP)‐Activin A Receptor Type 1 (ACVR1) signaling pathway is critical for the anti‐tumor efficacy of PRC2 inhibitors. The genes *BMP6*, *BMP7* and *ACVR1* are directly repressed by PRC2 in lymphoma cells. BMP‐ACVR1 signaling, mediated through SMAD1/5 phosphorylation, is activated by PRC2 inhibition or depletion. ACVR1‐deficiency significantly reduces the sensitivity of lymphoma to PRC2 inhibitors both in vitro and in vivo. Moreover, induced expression or application of BMP7 in lymphoma cells results in decreased proliferation. High levels of BMP6, BMP7 or ACVR1 are associated with significantly better survival in a human DLBCL cohort. Currently, there are no biomarkers to available to guide the clinic use of tazemetostat and other PRC2 inhibitors, limiting their application. The resistance mechanism potentially hindering their clinical efficacy remains unknown. Our study establishes a strong mechanistic and functional link between ACVR1 signaling and PRC2, suggesting that the BMP‐ACVR1 pathway may serve as an efficacy biomarker for PRC2 inhibitory drugs in lymphoma patients.

## 2 Results

### 2.1 Combined Genome‐Wide CRISPR/Cas9 Screen and Transcriptome Profiling Uncovers Effectors of PRC2 Inhibition

**Figure 1:**
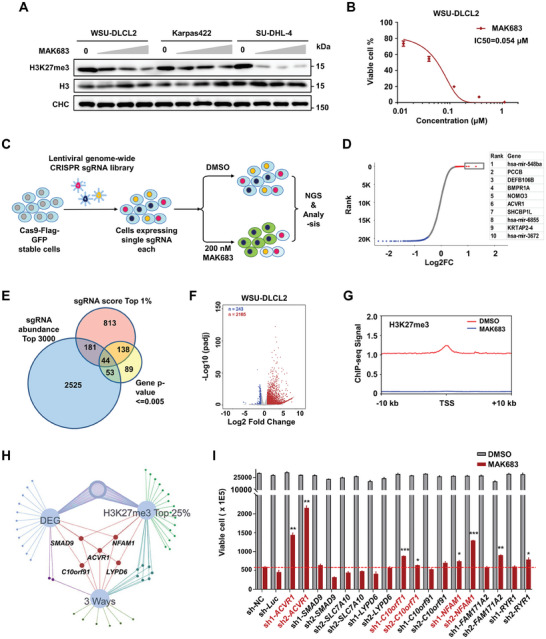
Combined CRISPR‐Cas9 screen and transcriptome profiling reveals the critical effectors of PRC2 inhibitor in lymphoma. A) Western blots showing modulation of H3K27me3 in WSU‐DLCL2, Karpas422 and SU‐DHL‐4 cells after incubation with MAK683 (0, 0.2, 1, and 5 µm) for 3 days. H3 and Clathrin heavy chain (CHC) were used as internal standards. B) The dose‐dependent inhibition of WSU‐DLCL2 proliferation by MAK683 after 13 days of treatment (*n* = 3, IC50 = 0.054 µm). C) Overview of the CRISPR‐Cas9 genome‐wide screening for resistance of PRC2 inhibitor in WSU‐DLCL2. D) Overview of gene distribution in the CRISPR screen ranked based on the log2 fold change to DMSO. The right table lists out the top 10 genes positively selected. E) Venn diagram of the three enrichment criteria to identify the top gene hits (sgRNA abundance top 3000, sgRNA score top 1%, and genes with *p*‐value ≤ 0.005. F) Volcano plot showing differentially expressed genes by RNA‐seq from WSU‐DLCL2 cells treated with MAK683 at 3 µm or DMSO for 3 days (*n* = 3 biologically independent samples). G) Composite H3K27me3 profile around transcripts start sites (TSS) in WSU‐DLCL2 cells treated with MAK683 at 3 µm or DMSO for 3 days. H) Venn diagram of three enrichment analysis (CRISPR‐screen list from E, DEG of RNA‐seq, and ChIP‐seq top 25%) to identify genes for further validation. I) Viable cells of WSU‐DLCL2 with individual indicated shRNAs after 12 days of treatment with DMSO or MAK683 at 0.05 µM (mean ± s.d., *n* = 2). Red line indicates viable cell of non‐targeting control (NC) shRNA with MAK683. Experiments were performed three times. *P* values were determined by two‐tailed unpaired t test (^*^, *p* < 0.05; ^**^, *p* < 0.01; ^***^, *p* < 0.001).

To systematically identify the functional effectors responsible for the anti‐lymphoma activity of PRC2 inhibitors, we conducted a proliferation‐based genome‐wide CRISPR/Cas9 knockout screen in the sensitive lymphoma cell WSU‐DLCL2. PRC2 inhibition by MAK683 led to a dose‐dependent reduction in global H3K27me3 levels in WSU‐DLCL2, Karpas422 and SU‐DHL‐4, all of which carry EZH2 Y641 mutations (**Figure** **1**A; Figure S1A, Supporting Information).^[^ ^10^ ^]^ WSU‐DLCL2 exhibited the highest sensitivity, with a half‐maximal inhibitory concentration (IC50) of 54 nM (Figure 1B). The Cas9 stable‐expressing WSU‐DLCL2 cell was established and transduced with a genome‐scale CRISPR knockout lentiviral pooled library (GeCKO v2) (Figure 1C; Figure S1B–D, Supporting Information).^[^ ^17^ ^]^ After 7 days of culture with puromycin selection, cells were subjected to treatment with DMSO or MAK683 (200 nM) for additional 14 days before sequencing (Figure 1C). This strategy ensured sgRNAs sensitizing the cell to PRC2 inhibition were depleted in the MAK683 treatment (negative selection), while sgRNAs targeting genes essential for the efficacy of MAK683 were enriched (positive selection) (Figure 1D). Notably, among the top 10 hits in positive selection, two BMP receptors, BMPR1A and ACVR1, were identified. Triaging the hit list using a 3‐way method resulted in the identification of 44 significantly positively selected genes, which also displayed a strong enrichment of BMP signaling and SMAD binding pathways in Gene Ontology (GO) analysis (Figure 1E; Figure S1E, Supporting Information).

As PRC2 plays a vital role in gene expression regulation through its histone H3K27 methyltransferase activity, we conducted RNA sequencing (RNA‐seq) and chromatin immunoprecipitation (ChIP)‐seq. MAK683 treatment led to the significant upregulation of >2000 genes, accompanied by a depletion of H3K27me3 signal, while H3K4me3 remained unaffected (Figure 1F,G; Figure S1F, Supporting Information). Integrated analysis of the CRISPR screen, RNA‐seq and ChIP‐seq data narrowed the validation list down to 9 genes (*ACVR1, SMAD9, SLC7A10, LYPD6, C10orf71, C10orf91, NFAM1, FAM171A2*, and *RYR1*) (Figure 1H). Subsequent validation using shRNAs revealed that knockdown of *ACVR1, C10orf71*, or *NFAM1* by two independent shRNAs impaired MAK683‐induced cell proliferation, with *ACVR1* emerging as the top effector (Figure 1I; Figure S1G, Supporting Information). Collectively, multiple lines of evidence converge on the BMP‐ACVR1 pathway, promoting us to focus on it in the following studies.

### 2.2 BMP‐ACVR1 Expression and Signaling are Epigenetically Regulated by PRC2 in DLBCL

The BMPs/Growth and Differentiation Factors subgroup of transforming growth factor‐β (TGF‐β) superfamily comprises over 20 secreted signaling factors that play crucial roles in embryonic development and adult tissue homeostasis.^[^ ^18^ ^]^ BMPs, as dimeric ligands, transmit signals through a hetero‐tetrameric receptor complex involving two type I and two type II transmembrane receptors, each with intrinsic serine‐threonine kinase activities. There are four type I receptors (BMPR1A, BMPR1B, ACVR1, and ACVRL1) and three type II receptors (BMPR2, ACVR2A, and ACVR2B), which can interact combinatorically. Upon signaling complex formation, the type II receptors phosphorylate and activate type I receptors, which in turn phosphorylate SMAD1/5/8. These phosphorylated SMADs, along with SMAD4, translocate to the nucleus to regulate target gene expression, such as *ID2* and *ID3*.^[^ ^18^ ^]^ *ACVR1* encodes the Activin A receptor type I (also called Activin receptor‐like kinase 2, ALK2). The ACVR1 protein can bind to Activin A, BMP2, BMP5, BMP6, BMP7, or BMP9 but not the other BMPs, and trigger SMAD1/5/8 signaling.^[^ ^18^ ^]^ Our data indicate that BMP‐ACVR1 signaling is not only required for the anti‐tumor efficacy of PRC2 inhibitor, but also repressed by PRC2 and upregulated upon PRC2 inhibition (Figure 1H). So, we further dissected the repression of BMP‐ACVR1 signaling by PRC2 in lymphoma.

**Figure 2:**
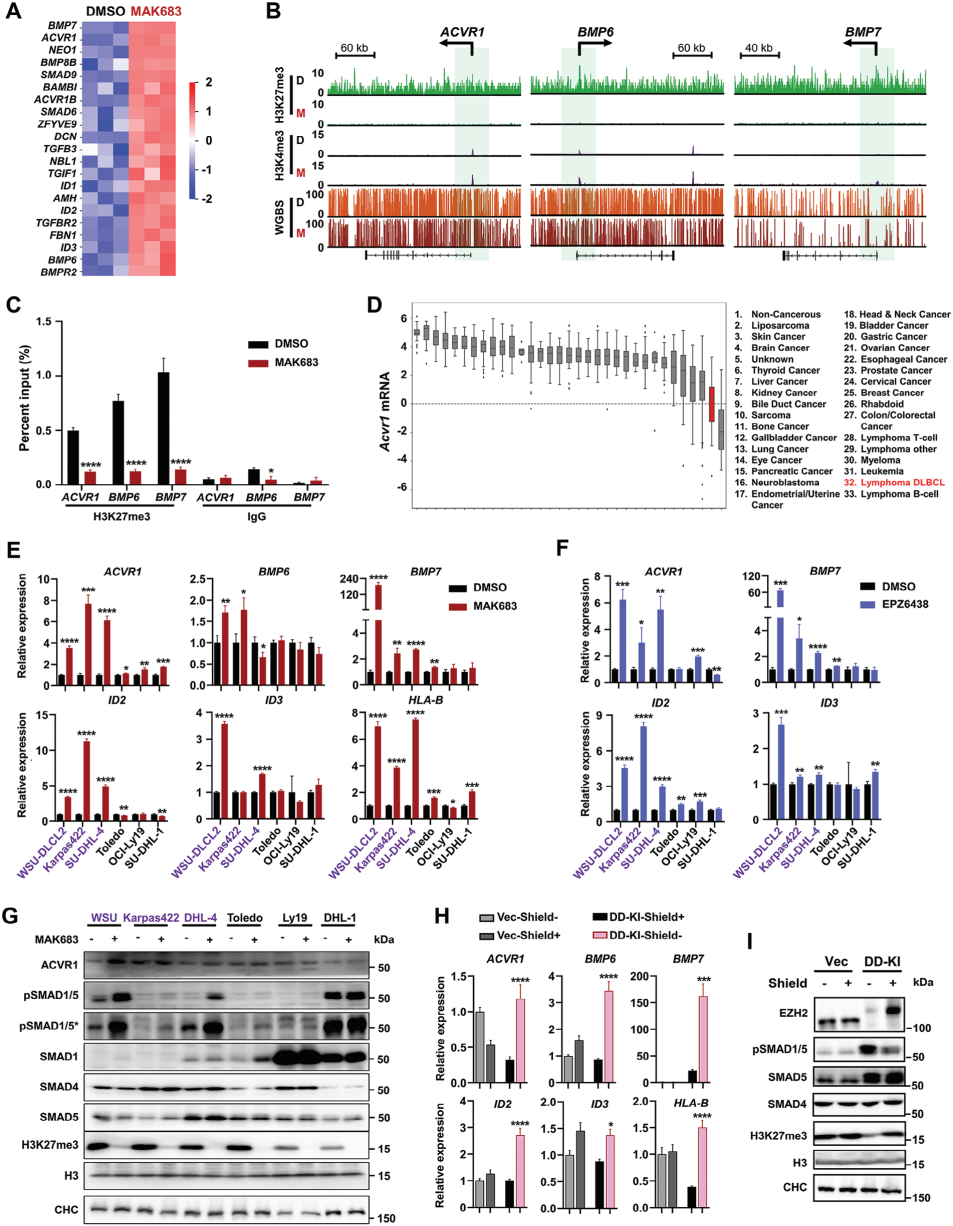
BMP‐ACVR1 expression and signaling are under tight epigenetic regulation of PRC2 in lymphoma cells. A) Heatmap showing RNA‐seq of TGF‐beta pathway genes in WSU‐DLCL2 treated with DMSO or MAK683 at 5 µm for 6 days (*n* = 3 biologically independent samples). B) H3K27me3 and H3K4me3 ChIP‐seq tracks and WGBS tracks at *ACVR1*, *BMP6*, and *BMP7* loci in WSU‐DLCL2 cells treated with DMSO or MAK683. Green boxes highlight genomic regions around TSS. C) ChIP‐qPCR of H3K27me3 in WSU‐DLCL2 at promotors of *ACVR1, BMP6, BMP7* after 6 days treatment of MAK683 or DMSO. Rabbit IgG was used as control (mean ± s.d., *n* = 3 biologically independent samples). D) *ACVR1* mRNA levels in different tumor types from CCLE (tumor types are indicated numerically from left to right, and red bar indicates lymphoma DLBCL). E) and F) RT‐qPCR of BMP‐ACVR1 pathway and canonical PRC2 target genes in WSU‐DLCL2, SU‐DHL‐4, Karpas422, OCI‐Ly19, Toledo and SU‐DHL‐1 cells treated with MAK683 (5 µm), EPZ6438 (5 µm) or DMSO for 4 days. All data were normalized to GAPDH and DMSO samples were arbitrarily set as 1 (mean ± s.d., *n* = 3). G) Western blots of ACVR1, SMADs proteins and H3K27me3 in WSU‐DLCL2, SU‐DHL‐4, Karpas422, OCI‐Ly19, Toledo and SU‐DHL‐1 cells treated with MAK683 (5 µm) or DMSO for 4 days. H,I) RT‐qPCR and western blots in Vector (Vec) and the C4 clone of EZH2‐DD‐KI (DD‐KI) cells. Vec and DD‐KI were in or withdrew from Shield treatment (1 µm) for 9 days. All data were normalized to GAPDH and Vec‐Shield‐ samples were arbitrarily set as 1 (mean ± s.d., *n* = 3). Experiments in C,E–I) were performed at least three times. Statistical analysis was performed using two‐tailed unpaired t tests for C,E,F) one‐way ANOVA for H) (^*^, *p* < 0.05; ^**^, *p* < 0.01; ^***^, *p* < 0.001; ****, *p* < 0.0001).

Through the differentially expressed genes (DEG) and GO analysis using RNA‐seq data from WSU‐DLCL2 lymphoma cell carrying *EZH2^Y641F^*, we observed an enrichment of TGF‐beta pathway with PRC2 inhibitor treatment, with *BMP7* and *ACVR1* as the top DEGs and multiple BMP ligands upregulated (**Figure** **2**A). Gene Set Enrichment Analysis (GSEA) also revealed a similar upregulation of the BMP‐ACVR1 signaling (Figure S2A, Supporting Information). In our H3K27me3 and H3K4me3 ChIP‐seq and Whole Genome Bisulfite Sequencing (WGBS) data, the genomic regions of *ACVR1, BMP6*, *and BMP7* exhibited high H3K27me3 and a positive H3K4me3 signature. MAK683 treatment efficiently removed H3K27me3 from these loci (Figure 2B). ChIP‐qPCR results also confirmed the loss of H3K27me3 on *ACVR1, BMP6*, *and BMP7* loci upon PRC2 inhibition by MAK683 (Figure 2C). So, PRC2 directly regulates the expression of BMP‐ACVR1 pathway genes, particularly *ACVR1, BMP6*, and *BMP7*, in WSU‐DLCL2 cell.

Next, we examined whether the regulation of *ACVR1* expression by PRC2 is a common phenomenon. We observed that *ACVR1* mRNA levels are low in lymphoma, particularly in B‐cell lymphoma and DLBCL, among all the cancer cells in Cancer Cell Line Encyclopedia (CCLE) (Figure 2D). This expression pattern of *ACVR1* showed a negative correlation with the high expression and activity of PRC2 in germinal center B cell (Figure S2B, Supporting Information).^[^ ^19^, ^20^ ^]^ In our expanded lymphoma panel, WSU‐DLCL2, Karpas422 and SU‐DHL‐4 carry the EZH2‐GoF mutations and are sensitive to PRC2 inhibition, while Toledo, OCI‐Ly19 and SU‐DHL‐1 do not and the H3K27me3 at *ACVR1* was lower in them (Figure S2C,D, Supporting Information). Upon PRC2 inhibition by MAK683 or EPZ6438, *BMP6*, *BMP7*, *ACVR1*, and the downstream targets *ID2* and *ID3* were uniformly upregulated in sensitive DLBCL cell lines (Figure 2E,F; Figure S2F, Supporting Information). *HLA‐B* and *p21* were known PRC2 targets and included as positive controls (Figure 2E; Figure S2E,F, Supporting Information).^[^ ^16^, ^21^ ^]^ BMP signaling leads to phosphorylation of SMAD1/5/8. Our western blot results with two different phospho‐SMAD1/5 (pSMAD1/5) antibodies confirmed the activation of BMP signaling upon PRC2 inhibition in the EZH2‐mutant cells (Figure 2G). To rule out the possibility of off‐target effects from compound treatment, an inducible EZH2 degradation cell line was constructed through CRISPR in‐frame knock‐in (KI) of a Shield compound‐stabilized degron (destabilizing domain, DD) at the N‐terminus of both EZH2 alleles (Figure S2G–I, Supporting Information, homozygous KI clone C4 was used in later studies).^[^ ^22^ ^]^ Withdraw of Shield led to the rapid degradation of DD‐EZH2, resulting in the significant upregulation of *BMP6*, *BMP7*, *ACVR1*, *ID2*, and *ID3* along with an increase in pSMAD1/5 (Figure 2H,I), consistent with the effects of MAK683 or EPZ6438 treatment.

Together, the removal of H3K27me3 marks through PRC2 inhibition or depletion results in the upregulation of *ACVR1* and the activation of BMP‐ACVR1 signaling, reflected by the phosphorylation of SMAD1/5 and the common upregulation of *ID2* or *ID3* in EZH2‐mutant lymphoma cells.

### 2.3 BMP‐ACVR1 Expression and Signaling are Upregulated by PRC2 Inhibitor In Vivo

**Figure 3:**
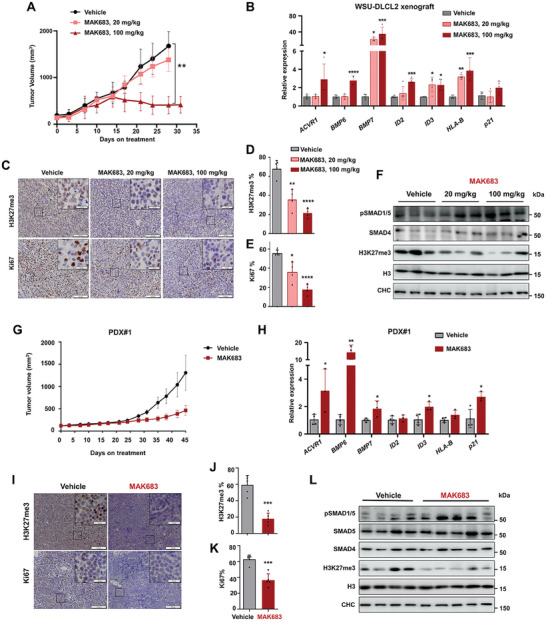
BMP‐ACVR1 expression and signaling are upregulated by PRC2 inhibitors in lymphoma in vivo. A) Growth curve of subcutaneous WSU‐DLCL2 xenograft tumors in mice treated with MAK683 or vehicle orally with indicated doses (mean ± s.e.m., *n* = 5). B) RT‐qPCR of indicated genes in tumor samples from the end point of study in (A). All data were normalized to GAPDH, and vehicle was arbitrarily set as 1 (mean ± s.d., *n* ≥ 4 mice per group). C–E), Representative H3K27me3 and Ki67 IHC images of tumor samples and the quantitated bar graphs (mean ± s.d., *n* = 4 mice per group) from the end point of study in (A). Scale bar for images, 100 µm; scale bar for the intersects, 20 µm. F) Western blots of SMADs and H3K27me3 in tumors from the end point of study in (A). G) Growth curve of subcutaneous lymphoma patient‐derived xenograft tumors (model ID: LD1‐0026‐362314, LIDE biotech) in mice treated with MAK683 (100 mg kg^−1^) or vehicle orally (mean ± s.e.m., *n* = 5). H) RT‐qPCR analysis of indicated genes in tumor samples from the end point of study in (G). All data were normalized to GAPDH, and vehicle was arbitrarily set as 1 (mean ± s.d., *n* ≥ 3 mice per group). I–K) Representative H3K27me3 and Ki67 IHC images of tumor samples and the quantitated bar graphs (mean ± s.d., *n* = 5 mice per group) from the end point of study in (G). Scale bar for images, 100 µm; scale bar for the intersects, 20 µm. L) Western blots of SMADs and H3K27me3 in tumors from the end point of study in (G). Experiments in (A–F) were performed at least two times. Statistical analysis was performed using two‐tailed unpaired t tests for H,J,K) one‐way ANOVA for A,B,D,E) (^*^, *p* < 0.05; ^**^, *p* < 0.01; ^***^, *p* < 0.001; ^****^, *p* < 0.0001).

PRC2 inhibition by EPZ6438 or MAK683 led to tumor regression in xenograft mouse models.^[^ ^23^, ^24^ ^]^ To investigate whether BMP‐ACVR1 signaling is activated by PRC2 inhibition in vivo, we examined the tumor xenograft of WSU‐DLCL2 with vehicle or oral dosing of MAK683. Dosing at 100 mg kg^−1^ led to significant tumor regression after 2 weeks without affecting body weight, while a low dose of 20 mg kg^−1^ mildly slowed tumor growth suggesting that 20 mg kg^−1^ is below effective dose (**Figure** **3**A; Figure S3A,B, Supporting Information). Consistent with the observations in cell culture, the genes in BMP‐ACVR1 pathway were broadly upregulated in tumors of the 100 mg kg^−1^ MAK683 group and to a lesser extent in the 20 mg kg^−1^ group (Figure 3B). Immuno‐histological chemistry (IHC) results confirmed a significantly decrease of H3K27me3 in the MAK683 groups and the proliferation marker Ki67 was also markedly reduced especially in the MAK683 100 mg kg^−1^ group, which correlated with the tumor size (Figure 3C–E). Although the pSMAD1/5 antibody is not suitable for IHC, we performed western blot and observed a notable increase of pSMAD1/5 signal, showing the activation of BMP‐ACVR1 signaling upon PRC2 inhibition in vivo (Figure 3F).

Taking one step further, we extended to two lymphoma patient‐derived xenograft (PDX) models, which are more clinically relevant than the cell‐derived xenograft. The first PDX model carries no GoF mutation but only wild‐type *EZH2* (LIDE Biotech, model ID: LD1‐0026‐362314). The treatment with MAK683 inhibited tumor growth without affecting body weight (Figure 3G; Figure S3C, Supporting Information). Meanwhile, the tumor samples exhibited a dramatic increase in pSMAD1/5 and the expression of BMP pathway genes along with a reduction in Ki67 in IHC of the xenograft tumor samples (Figure 3H–L). HJM‐353 is an EED binder/inhibitor that employs the same PRC2 inhibitory mechanism as MAK683 and exhibits good potency and selectivity.^[^ ^5^ ^]^ HJM‐353 dosing in vivo was applied in the second PDX model with wild‐type *EZH2* (Crown Bioscience, model# LY6934), and an enhancement of *ACVR1* expression and signaling through pSMAD1/5 was observed (Figure S3D–G, Supporting Information). Analysis of the RNA‐seq data from these tumor samples revealed an upregulation of BMP pathways by HJM‐353 treatment (Figure S3H,I, Supporting Information). In summary, our data demonstrate that PRC2 inhibition or deletion results in the upregulation and activation of BMP‐ACVR1 signaling through phosphorylation of SMAD1/5 in multiple PRC2 drug‐sensitive lymphoma cells and xenograft tumors.

### 2.4 Blocking BMP‐ACVR1 Signaling Compromises the Anti‐Proliferation of PRC2 Inhibition or Depletion

**Figure 4:**
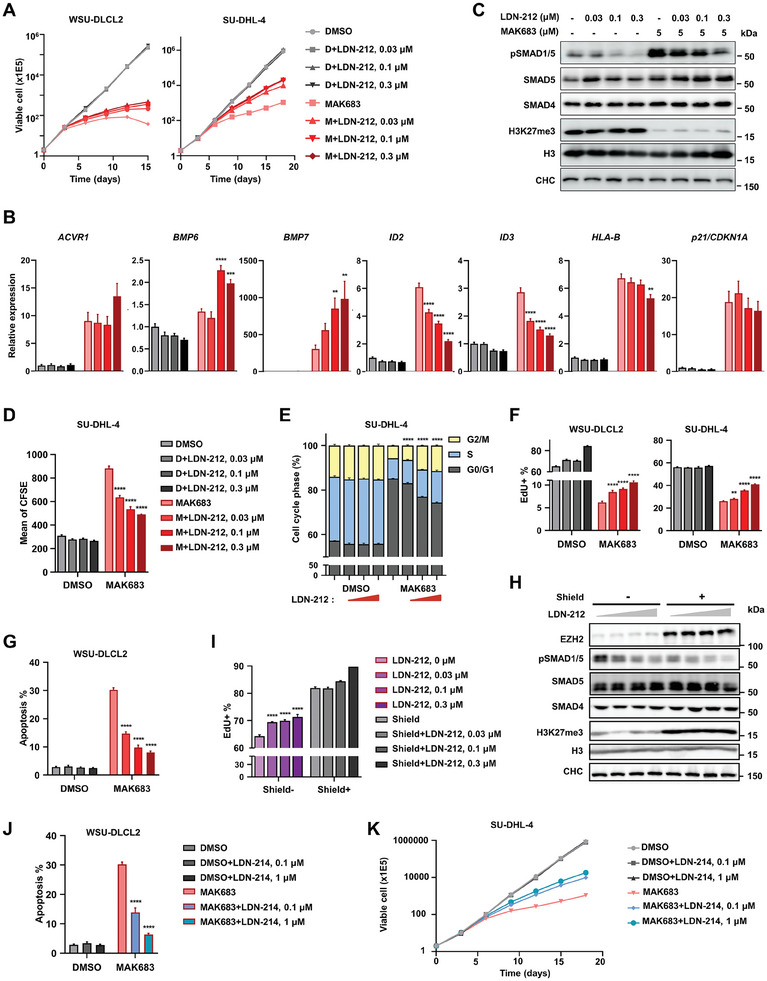
ACVR1 signaling promotes G1 arrest and apoptosis and facilitates the anti‐lymphoma activity of PRC2 inhibitors. A) Proliferation of WSU‐DLCL2 and SU‐DHL‐4 cells treated with MAK683 (1.2 µM) and/or LDN‐212854 at the indicated concentrations. Viable cells were counted every 3 days (mean ± s.d., *n* = 2). B,C) RT‐qPCR analysis of the indicated genes and western blot analysis of SMADs and H3K27me3 in WSU‐DLCL2 cells treated with MAK683 (5 µM) and/or LDN‐212854 at the indicated concentrations for 6 days. All data were normalized to GAPDH and DMSO was arbitrarily set as 1 (mean ± s.d., *n* = 3) in (B). D) Statistic description of CFSE in SU‐DHL‐4 cells treated with MAK683 (5 µm) and/or LDN‐212854 at the indicated concentrations for 10 days (mean ± s.d., *n* = 4). E) Cell cycle analysis of SU‐DHL‐4 cells treated with MAK683 (5 µm) and/or LDN‐212854 at the indicated concentrations for 9 days (mean ± s.d., *n* = 3). F) EdU incorporation analysis of WSU‐DLCL2 and SU‐DHL‐4 cells treated with MAK683 (5 µm) and/or LDN‐212854 at the indicated concentrations for 6 or 12 days respectively (mean ± s.d., *n* ≥ 3). G) Cell apoptosis in WSU‐DLCL2 cells treated with MAK683 (5 µm) and/or LDN‐212854 at the indicated concentrations for 9 days, determined by sub G1‐content from PI staining and FACS (mean ± s.d., *n* = 4). H,I) Western blots and EdU incorporation analysis (mean ± s.d., n = 3) of the C4 clone of EZH2‐DD‐KI (DD‐KI) cells treated with shield (0.5 µm) and/or LDN‐212854 for 9 days. J) Cell apoptosis in WSU‐DLCL2 cells treated with LDN‐214117 and/or MAK683 (5 µm) for 9 days, determined by sub G1‐content from PI staining and FACS (mean ± s.d., *n* = 4). K) Proliferation of SU‐DHL‐4 cells treated with LDN‐214117 and/or MAK683 (1.2 µm). Viable cells were counted every 3 days (mean ± s.d., *n* = 2). All cellular experiments were performed at least two times and representative data are shown here. Statistical analysis was performed using one‐way ANOVA (^*^, *p* < 0.05; ^**^, *p* < 0.01; ^***^, *p* < 0.001; ^****^, < 0.0001).

As we initially discovered ACVR1 and BMPR1A as the top hits among the survivors from PRC2 inhibitor CRISPR screen (Figure 1D), blocking ACVR1 signaling should compromise the anti‐proliferative effect of PRC2 inhibitor. To investigate this further, we utilized a chemical inhibitor LDN‐212854 (LDN‐212 for short thereafter), which inhibits BMP type I receptor kinases and shows specificity for ACVR1 over the others.^[^ ^25^ ^]^ MAK683‐induced proliferation inhibition was indeed partially blocked by LDN‐212 in both WSU‐DLCL2 and SU‐DHL‐4 cells (**Figure** **4**A). LDN‐212 reduced pSMAD1/5 and downregulated *ID2* and *ID3* in a dose‐dependent manner but it did not affect H3K27me3 erasure or the upregulation of *ACVR1, HLA‐B*, and *p21/CDKN1A* induced by MAK683 (Figure 4B,C; Figure S4A,B, Supporting Information). Additionally, the expression of *BMP6/7* were further upregulated in response to LDN‐212 treatment, suggesting feedback autoregulation.

Next, we dissected the effects of ACVR1 inhibition on cell division, cell cycle and apoptosis, as they may contribute to the observed changes in cell proliferation. Carboxyfluorescein succinimidyl ester (CFSE) pulse‐chase labeling showed a faster cell division with LDN‐212 treatment, which might be driven partially by less cell cycle arrest at G_0_/G_1_ (Figure 4D,E; Figure S4C–E, Supporting Information). Consistently, 5‐Ethynyl‐2′‐deoxyuridine (EdU) labeled increase of cells undergoing DNA synthesis with LDN‐212 dose‐dependently in the presence of MAK683 (Figure 4F). Less sub‐G_1_ apoptotic population was observed with LDN‐212 under MAK683 treatment, especially in WSU‐DLCL2 (Figure 4G; Figure S4F, Supporting Information). Utilizing the DD‐EZH2 KI cell,^[^ ^22^ ^]^ we confirmed that LDN‐212 reverses cell cycle block induced by PRC2 depletion (Figure 4H,I). Comparing with LDN‐212, LDN‐214117 (LDN‐214 for short) is a more selective kinase inhibitor of ACVR1.^[^ ^26^ ^]^ LDN‐214 antagonized the cell cycle arrest and apoptosis‐inducing effects of MAK683 similarly as LDN‐212 (Figure 4J,K; Figure S4G–I, Supporting Information). Thus, we used two different ACVR1 inhibitors to reassure blocking ACVR1 signaling partially neutralized the anti‐lymphoma effect of MAK683. Together, PRC2 inhibition or depletion leads to the activation of BMP‐ACVR1 signaling, which contributes to the cell cycle arrest, apoptosis, and proliferation blockade in lymphoma cells. Although it would be beneficial to include LDN‐212 or LDN‐214 in an in vivo study especially in PDX models, we used a genetic approach for in vivo validation of the role of ACVR1 signaling.

### 2.5 *ACVR1* Deletion Enables the Resistance to PRC2 Inhibitors in B Cell Lymphoma

**Figure 5:**
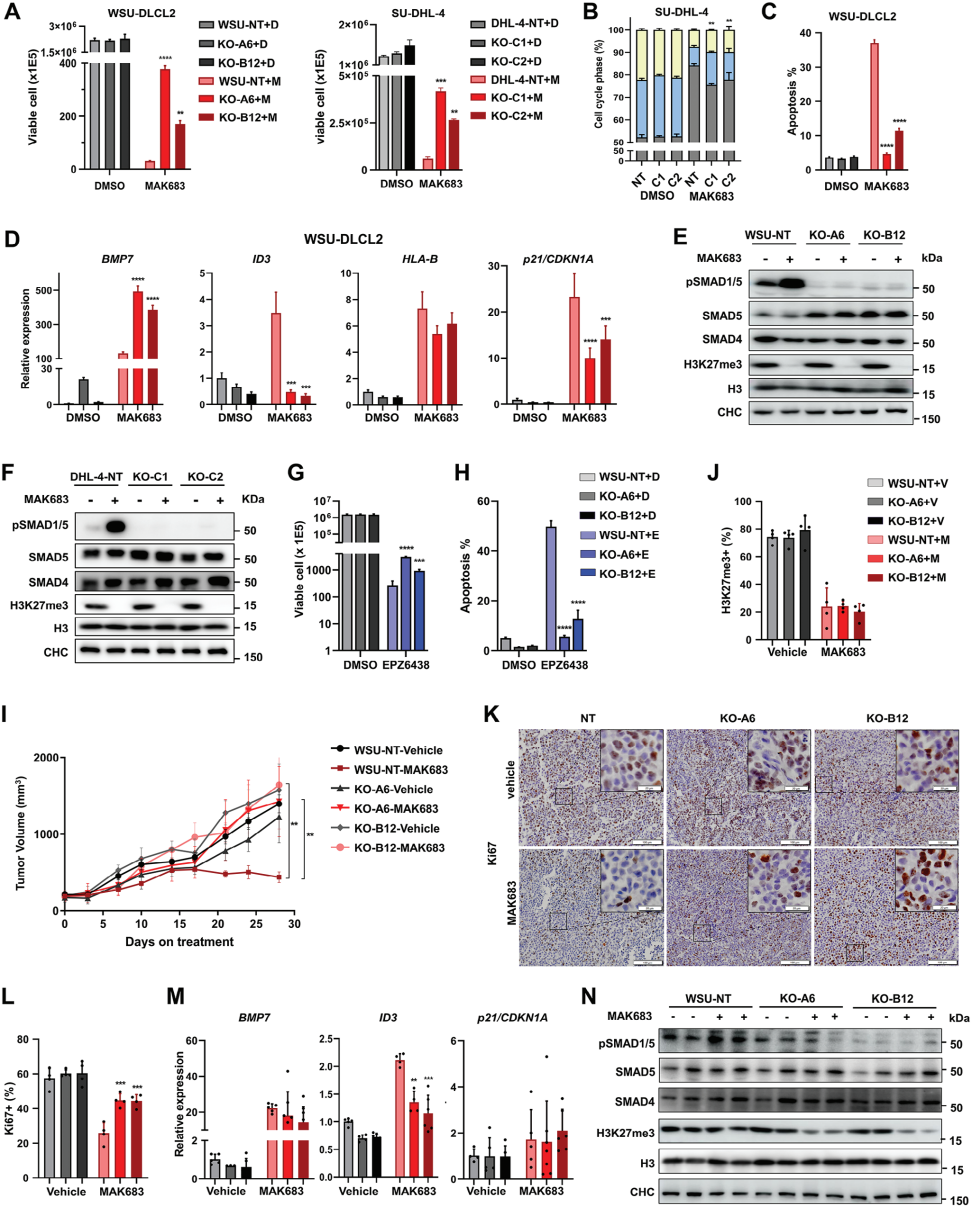
ACVR1 deletion compromises the anti‐lymphoma activity of PRC2 inhibitors. A) Viable cells of WSU‐DLCL2 and SU‐DHL‐4 cell clones treated with DMSO or MAK683 (1.2 µm) for 18 days. NT, a non‐targeting sgRNA control clone; A6, B12, C1, and C2, confirmed *ACVR1* knockout clones by DNA sequencing (mean ± s.d., *n* = 2). B) Cell cycle analyzed by FACS of SU‐DHL‐4 (NT, C1, C2) cells treated with MAK683 (5 µm) or DMSO for 9 days (mean ± s.d., *n* = 3). C) Cell apoptosis in NT, A6 and B12 clone cells treated with MAK683 (5 µm) or DMSO for 9 days, determined by sub G1‐content from PI staining and FACS (mean ± s.d., *n* = 4). The bars are color‐coded as in (A). D)‐F) RT‐qPCR analysis of indicated genes and western blots of indicated proteins in WSU‐DLCL2 (NT, A6, B12) and SU‐DHL‐4 (NT, C1, C2) cells treated with DMSO or MAK683 (5 µm) for 6 days. In (D), all data were normalized to GAPDH and NT‐DMSO samples were arbitrarily set as 1, and the bars are color‐coded as in (A) (mean ± s.d., *n* = 3). G) Viable cells in NT, A6 and B12 cells treated with DMSO or EPZ6438 (1.2 µm) for 18 days (mean ± s.d., *n* = 3). H) Cell apoptosis in NT, A6 and B12 cells treated with EPZ6438 (5 µm) or DMSO for 9 days, determined by sub G1‐content from PI staining and FACS (mean ± s.d., *n* = 3). I) Growth curve of subcutaneous NT, A6 and B12 xenograft tumors in mice treated with MAK683 (100 mg kg^−1^) or vehicle orally twice a day (mean ± s.e.m., *n* = 5). J) The percentage of H3K27me3 positive cells in tumor samples (mean ± s.d., *n* = 4 mice per group). K) Representative Ki67 IHC images of tumor samples from the end point of study in (I). Scale bar for images, 100 µm; scale bar for the intersects, 20 µm. L) The percentage of Ki67 positive cells in tumor samples (mean ± s.d., *n* = 4 mice per group). M,N) RT‐qPCR analysis of the indicated genes and western blots of SMADs and H3K27me3 in tumor samples from panel (I). All data were normalized to GAPDH, and NT‐vehicle samples were arbitrarily set as 1 (mean ± s.e.m., *n* ≥ 3). The cellular experiments were performed at least three times, and the xenograft study was performed at least two times. Statistical analysis was performed using one‐way ANOVA (^*^, *p* < 0.05; ^**^, *p* < 0.01; ^***^, *p* < 0.001; ^****^, < 0.0001).

In parallel to the ACVR1 inhibitor studies, we designed sgRNAs, constructed the *ACVR1* KO clones in WSU‐DLCL2 and SU‐DHL‐4, and proceeded with detailed analysis for two independent clones each. The *ACVR1* KO clones from WSU‐DLCL2 were named A6 and B12, and the KO clones from SU‐DHL‐4 were named C1 and C2. They all exhibited bi‐allelic disruption and expressed little to no *ACVR1* in qPCR analysis (Figure S5A–C, Supporting Information). *ACVR1* KO clones exhibited faster proliferation, reduced cell cycle arrest, and significantly less apoptosis compared with non‐targeting control cells (NT) under MAK683 treatment (**Figure** **5A–C**; Figure S5D–G, Supporting Information). These phenotypic effects of *ACVR1* KO mirrored the effects of ACVR1 chemical inhibitors (Figure 4). Not surprisingly, the enhanced pSMAD1/5 signal or upregulation of *ID2/3* upon MAK683 treatment were completely absent in *ACVR1* KO clones (Figure 5D–F; Figure S5H, Supporting Information). The improvement in proliferation and cell viability by *ACVR1* KO was also observed when PRC2 was inhibited by EPZ6438 (Figure 5G,H), suggesting that *ACVR1* is essential to enable the full sensitivity to PRC2 inhibitors regardless of the chemical structure or binding mode.

The *ACVR1* KO clones provided us with tools to assess the effect of blocking BMP‐ACVR1 pathway in vivo. Both *ACVR1* KO clones exhibited similar growth kinetics as WSU‐DLCL2 NT cells in xenograft over 28 days. However, MAK683 only effectively inhibited the NT tumors, but not the *ACVR1* KO‐A6 or ‐B12 tumors (Figure 5I; Figure S5I, Supporting Information). Examination of IHC slices with indicated antibodies suggested that MAK683 similarly decreased the H3K27me3 in all three xenograft tumors, but the proliferation marker Ki67 was decreased much more dramatically in NT tumors compared to A6 and B12 tumors (Figure 5J–L; Figure S5J, Supporting Information). qPCR and Western blot analysis confirmed the deficiency in SMAD1/5 phosphorylation and *ID3* expression despite the BMP ligands, *p21/CDKN1A* and *HLA‐B* were similarly upregulated by PRC2 inhibition (Figure 5M,N; Figure S5K, Supporting Information), although a small residue level of H3K27me3 (≈25–30%) was observed (Figure 5J,N; Figure S5J, Supporting Information). Thus, *ACVR1* deletion enables resistance to PRC2 inhibitors in lymphoma.

### 2.6 BMP‐ACVR1 Signaling is Required for Efficient Upregulation of B Cell Differentiation by PRC2 Inhibition

**Figure 6:**
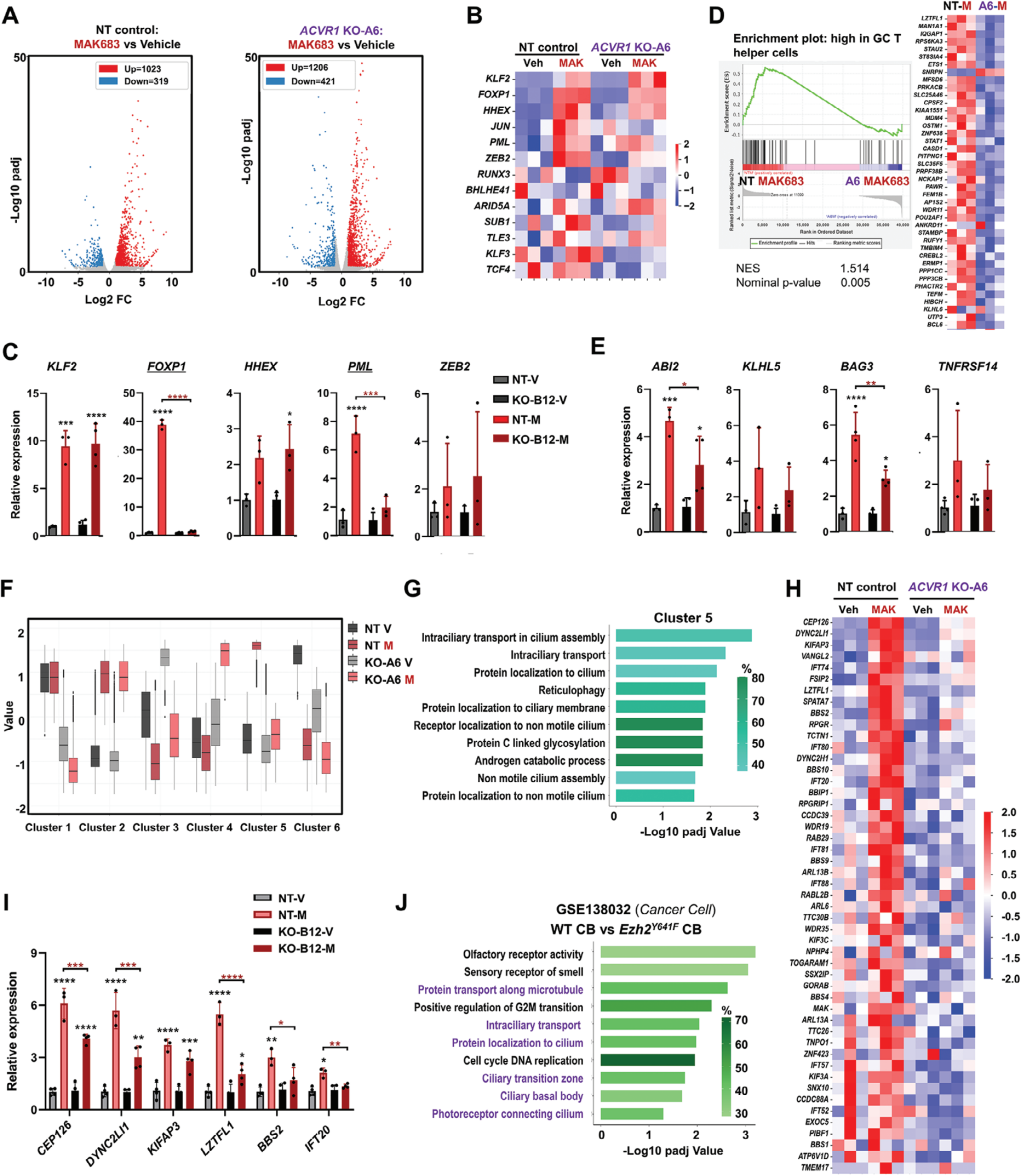
ACVR1 is required for MAK683‐induced upregulation of immunological synapse. A) Volcano plots showing the differentially expressed genes (DEGs) in xenograft tumors with dosing of MAK683 or vehicle in Figure 5I (*n* = 3 independent tumors). Red indicates upregulation with MAK683 (log2 fold change ≥ 1 and *P* adjust ≤ 0.05) and blue indicates downregulation with MAK683 (log2 fold change ≤ −1 and *P* adjust ≤ 0.05). B) Heatmap of RNA‐seq data from (A) for a panel of transcriptional factors in memory B cell differentiation. C) RT‐qPCR of the indicated genes in tumors with dosing of MAK683 or vehicle in Figure 5I. All data were normalized to GAPDH, and vehicle samples were arbitrarily set as 1 (mean ± s.d., *n* = 3). D) GSEA enrichment of the indicated gene set and heatmap of RNA‐seq data for the gene set. E) RT‐qPCR of the indicated genes as in *c* (mean ± s.d., *n* = 3). F) Box plot of log2 fold change in the 6 gene clusters from unsupervised clustering analysis of RNA‐seq data. The whiskers of the box plot extend to 1.5 times the interquartile range. G) The top enriched GO pathways in the cluster 5 from (F). H) Heatmap depicting the gene expression in RNA‐seq data for the core set of genes from pathways in (G). I) RT‐qPCR analysis of the indicated genes in tumor samples from study in Figure 5I. All data were normalized to GAPDH, and vehicle samples were arbitrarily set as 1 (mean ± s.d., *n* ≥ 3). J) GSEA analysis of upregulated genes in WT versus *Ezh2^Y641F^* centroblasts (RNA‐seq data from GSE138032). Experiments in (C,E,I) were performed two times. Statistical analysis was performed using one‐way ANOVA (^*^, *p* < 0.05; ^**^, *p* < 0.01; ^***^, *p* < 0.001; ^****^, < 0.0001). Black asterisks indicate the difference between MAK683 and vehicle. Red asterisks indicate the difference between NT and *ACVR1* KO‐B12 with MAK683.

It is particularly interesting to observe that the deletion of *ACVR1*, which is regulated by PRC2, almost completely abolished the anti‐tumor efficacy of PRC2 inhibitors in xenograft model (Figure 5I). So, we performed RNA‐seq and analysis on the xenograft tumor samples. First, the overall patterns of transcriptome change induced by MAK683 were similar in NT and *ACVR1* KO‐A6 tumors (**Figure** **6**A). As we and others have shown PRC2 inhibition induces B cell differentiation,^[^ ^23^, ^24^ ^]^ we checked a small panel of transcription factors critical for memory B cell differentiation.^[^ ^27^ ^]^ Indeed, *KLF2*, *FOXP1*, *HHEX*, *PML*, and *ZEB2* were upregulated by MAK683 not only from RNA‐seq data but also validated by qPCR (Figure 6B,C). Especially, *FOXP1* and *PML* showed mis‐regulation in *ACVR1* KO tumors, suggesting ACVR1 signaling contributes to the memory B cell differentiation. Considering PRC2 was similarly inhibited by MAK683 but the response of NT and *ACVR1* KO‐A6 tumors was different, we also examined the differentially expressed pathways between NT‐MAK683 and *ACVR1* KO‐A6‐MAK683. GSEA analysis identified the “GC T‐helper up genes” enriched in NT‐MAK683 and many genes in this gene set are involved in B cell and T‐helper cell interaction in GC (Figure 6D). This observation is consistent with the results from a prior study using *Ezh2^Y641F^* knock‐in mouse model, which showed that EZH2^Y641F^ centrocytes manifested a decreased interaction/dependency on T‐helper cell in GC reaction.^[^ ^8^ ^]^ Following this vein, a few genes involved in T‐helper cell interaction and centrocyte recycling to dark zone^[^ ^8^ ^]^ were examined and they showed a trend of upregulation with PRC2 inhibitor, while *ACVR1* KO tended to compromise this upregulation (Figure 6E).

To further dissect the genes differentially regulated in NT comparing with *ACVR1* KO, we performed an unsupervised clustering analysis (Figure 6F). Among the six differently modulated clusters of genes, the cluster 5 uniquely exhibited upregulation by MAK683 in NT but was blocked in *ACVR1* KO, while cluster 2 genes were upregulated by MAK683 regardless of ACVR1. In GO analysis, Interferon Gamma or Antigen Presentation related pathways were enriched in cluster 2, which has been identified previously (Figure S6A, Supporting Information).^[^ ^21^ ^]^ On the other hand, the top enriched pathways in cluster 5 were related to cilium assembly and intraciliary transport (Figure 6G). In the leading‐edge core genes from cluster 5, many of them were upregulated by PRC2 inhibition, while this upregulation was diminished in *ACVR1* KO xenograft tumors in both RNA‐seq and qPCR validation (Figure 6H,I). Among the 1023 upregulated DEGs, 299 genes ae dependent on ACVR1 (cluster 5, Figure 6A,F). Applying the same analysis to RNA‐seq data from *Ezh2^Y641F^* knock‐in GC B cells (GSE138032), we found a similar enrichment of cilia related pathways in WT versus *Ezh2^Y641F^* B cells (Figure 6J; Figure S6B–F, Supporting Information). Although there are no cilia in lymphocytes, many reports support that cilia proteins, such as BBS1 and LZTFL1, contribute to the dynamic assembly of immune synapse and signaling in lymphocytes.^[^ ^28^, ^29^ ^]^ So they may be required for the efficient Tfh interaction and affinity selection of GCB cell. In summary, immune synapse‐related genes are upregulated by PRC2 inhibition in responsive lymphomas and ACVR1 signaling strengthened this upregulation. The lack of upregulation of these genes can be enabled by *ACVR1* depletion and may associate with PRC2 inhibitor resistance (Figures 5 and 6).

### 2.7 BMP‐ACVR1 Signaling is a Tumor‐Suppressive Pathway in B‐Cell Lymphoma

Our results clearly demonstrated the regulation of BMP‐ACVR1 by PRC2 in B‐cell lymphoma and this signaling is critically required for the anti‐lymphoma efficacy of PRC2 inhibitors. One typical feature of BMP pathway is its promiscuity.^[^ ^30^, ^31^, ^32^ ^]^ There are >20 BMP family ligands competing for the limited type I and type II receptors.^[^ ^30^, ^31^, ^32^ ^]^ Conversely, each ligand could utilize multiple receptor proteins. So, the receptor tetramer complex for a specific BMP ligand may be tissue‐ or developmental stage‐dependent.^[^ ^32^ ^]^ We next examined the BMP ligands and receptor complex in B‐cell lymphoma.

**Figure 7:**
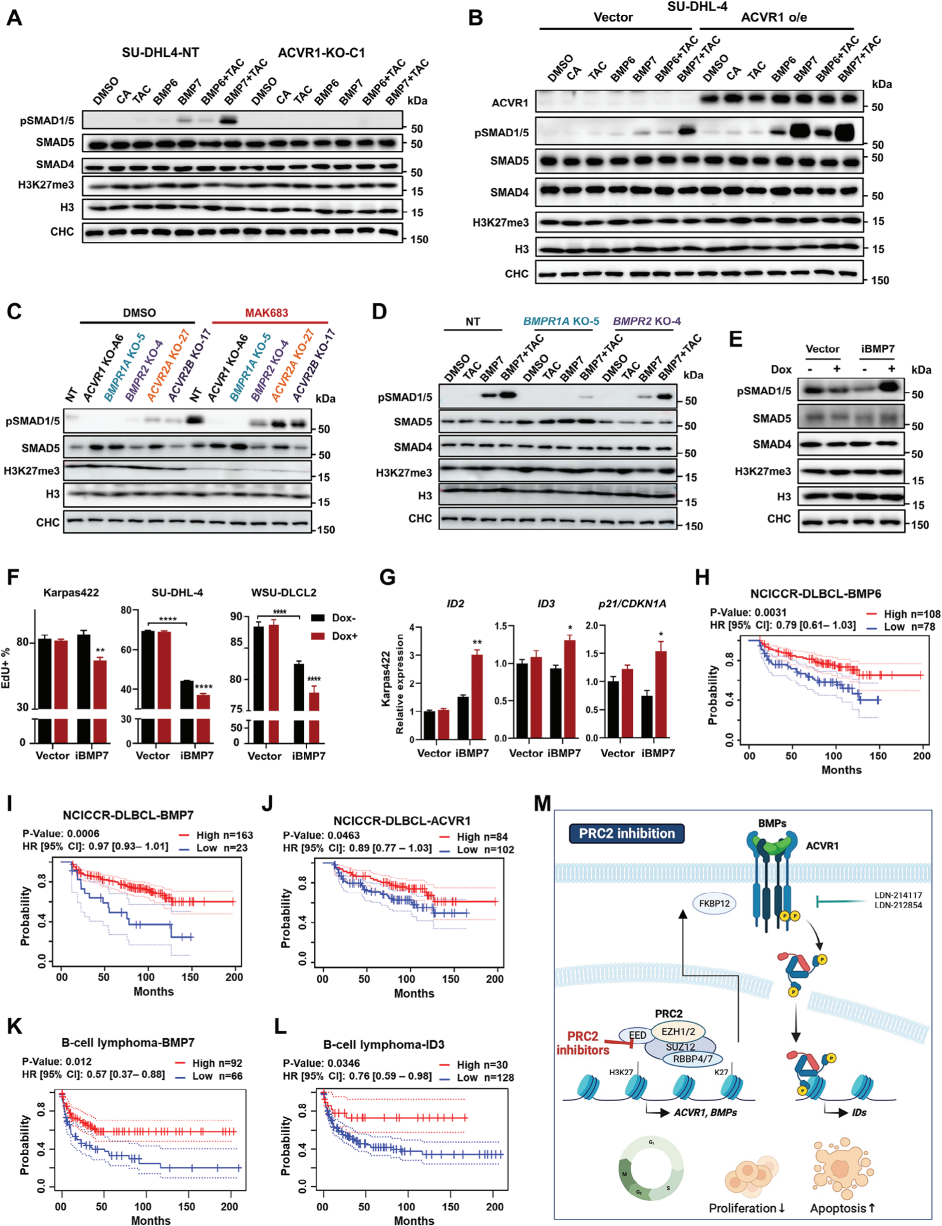
BMP‐ACVR1 signaling is a tumor‐suppressive pathway in B‐cell lymphoma. A,B) Western blots of pSMAD1/5 or the indicated proteins in SU‐DHL4 cells treated with DMSO, Cyclosporin A (CA, 1 µg mL^−1^), Tacrolimus (TAC, 1 µg mL^−1^), BMP6 (100 ng mL^−1^) and/or BMP7 (100 ng mL^−1^) for 45 min after 20 h starvation. C) Western blots of pSMAD1/5 or the indicated proteins in the individual knockout clone cells of WSU‐DLCL2 treated with DMSO or MAK683 (5 µm) for 3 days. D) Western blots of pSMAD1/5 or the indicated proteins in NT, BMPR1A KO‐5 and BMPR2 KO‐4 clones treated with DMSO, Tacrolimus (1 µg mL^−1^) and/or BMP7 (100 ng mL^−1^) for 45 min after 20 h starvation. E) Western blot analysis of BMP‐ACVR1 signaling in iBMP7‐Karpas422 cells treated with or without doxycycline (500 ng mL^−1^) treatment for 2 days. F) EdU incorporation analysis of iBMP7‐SU‐DHL‐4, iBMP7‐Karpas422 and iBMP7‐WSU‐DLCL2 cells treated with or without doxycycline (500 ng mL^−1^) for 6 days (mean ± s.d., *n* = 3). G) RT‐qPCR analysis of indicated genes in iBMP7‐Karpas422 cells with or without doxycycline (500 ng mL^−1^) treatment for 2 days. All data were normalized to GAPDH, and vehicle samples were arbitrarily set as 1 (mean ± s.d., *n* = 3). H–J) Kaplan–Meier survival curve of DLBCL patients stratified based on *BMP6, BMP7*, or *ACVR1* expression level. Data are from NCICCR cohort (*n* = 108 and *n* = 78 for *BMP6* hi and lo/int, *n* = 163 and *n* = 23 for *BMP7* hi and lo/int, *n* = 84 and *n* = 102 for *ACVR1* hi and lo/int, respectively). K,L) Kaplan–Meier survival curves of B cell lymphoma patients stratified based on *BMP7* or *ID3* expression level (GSE4475). Data are from PrognoScan cohort (*n* = 92 and *n* = 66 for *BMP7* hi and lo/int, *n* = 30 and *n* = 128 for *ID3* hi and lo/int, respectively). M) A proposed model: PRC2 inhibitors de‐repress BMP‐ACVR1 expression and signaling to reenforce their anti‐lymphoma efficacy. All cellular experiments were performed at least three times. Statistical analysis was performed using two‐tailed unpaired t test (^*^, *p* < 0.05; ^**^, *p* < 0.01; ^***^, *p* < 0.001; ^****^, < 0.0001).

*BMP6* and *BMP7* are upregulated by PRC2 inhibition or depletion in lymphoma cells (Figure 2) and we purchased and applied them individually. As the activation of type I receptors of BMP family is inhibited by FKBP12, which binds to the GS rich region of the ligand‐free type I receptors,^[^ ^33^ ^]^ we also included Tacrolimus (TAC), the blocker of FKBP12 in combination with BMP. The endogenous ACVR1 responded to BMP6 or BMP7 and promoted the SMAD1/5 phosphorylation, which required the presence of ACVR1 (**Figure** **7**A; Figure S7A, Supporting Information). Overexpressing ACVR1 or blocking FKBP12 by TAC only enhanced the signaling through pSMAD1/5 in the presence of BMP6 or BMP7. BMP7 induced stronger pSMAD1/5 than BMP6 (Figure 7A,B; Figure S7A,B, Supporting Information).

For BMP receptors, the expression of *BMPR1B* and *ACVRL1* was barely detectable in lymphoma cells (Figure S7C, Supporting Information). So, we focused on *BMPR1A*, *BMPR2*, *ACVR2A*, and *ACVR2B*, designed the sgRNAs targeting them individually and constructed the KO cells (Figure S7D, Supporting Information). Consistent with our original sgRNA screening result, *BMPR1A* KO strongly blocked the pSMAD1/5 to the same extend as *ACVR1* KO (Figure 7C). The depletion of *BMPR2* only partially decreased the pSMAD1/5 signal in response to MAK683, while *ACVR2A* or *ACVR2B* KO did not affect the pSMAD1/5 level (Figure 7C). Indeed, BMP7‐induced SMAD1/5 phosphorylation was blocked in *BMPR1A* KO cell and only partially diminished in *BMPR2* KO cells (Figure 7D). Therefore, type I receptors ACVR1 and BMPR1A are both required for the enhanced BMP signaling upon PRC2 inhibition, and BMPR2 is also involved as a type II BMP receptor.

As ACVR1 expression is low in lymphoma and PRC2 blocks its signaling, could artificially activating BMP‐ACVR1 signaling block lymphoma? We next constructed the inducible BMP7 expressing cells to examine the effect of chronic BMP signaling activation since the BMP7 upregulation by PRC2 inhibitor ranked top (Figure 2A). Although the *BMP7* mRNA increase was mild, we observed a significant signaling cascade activation and a decrease in EdU labelling upon BMP7 induction in WSU‐DLCL2, Karpas422 and SU‐DHL‐4, indicating that the BMP7‐ACVR1 signaling represses the proliferation of lymphoma cells (Figure 7E–G; Figure S7E,F, Supporting Information).

It has been well established that the level of EZH2 correlates tightly with poor patient survival in many cancers.^[^ ^34^, ^35^ ^]^ How about the BMP‐ACVR1 pathway? We performed the survival analysis of BMP ligands and receptors in the cohort of NCICCR‐DLBCL patients (*n* = 186).^[^ ^36^ ^]^ The levels of BMP6, BMP7 and ACVR1 significantly correlated with better survival outcome probability (Figure 7H–J), especially the level of BMP7 (P value = 0.0006). BMPR1A, on the contrary, showed no significant correlation (Figure S7G, Supporting Information). In another cohort of B‐cell lymphoma patients,^[^ ^37^ ^]^ BMP7 or ID3 high also significantly associated with better survival (Figure 7K,L). Together, BMP‐ACVR1 signaling is a tumor suppressive pathway in B‐cell lymphoma, as it inhibits lymphoma cell proliferation and significantly correlates with better survival in patients.

## 3 Discussion

PRC2 inhibitor tazemetostat is approved for the treatment of FL and is in clinical trials of DLBCL (NCT05618366 and NCT05604417). There are other PRC2 inhibitors in Ph2 or Ph3 anti‐tumor trials (MAK683, CPI‐1205, SHR2554, PF‐06821497, etc.). This study revealed a pharmacological mechanism of PRC2 inhibitors, which can be critical for their appropriate clinical application. First, *ACVR1* and *BMPR1A* were top hits of the CRISPR screen for WSU‐DLCL2 cell survival with MAK683 treatment. Second, PRC2 represses the expression of *BMP6*, *BMP7*, and *ACVR1* by H3K27me3 in B cell lymphoma in vitro and in vivo. Third, ACVR1 inhibition or deletion abolished the anti‐lymphoma effect of PRC2 inhibitors in cell culture and xenograft tumors, likely through blocking differentiation and immunological synapse‐mediated signaling in lymphoma. Finally, the high levels of *BMP6*, *BMP7*, and *ACVR1* correlate with a favorable survival outcome in B‐cell lymphoma patients. Together, this study identifies and demonstrates that BMP6/7‐ACVR1 signaling is a downstream pathway of PRC2 and plays an essential role in the efficacy of PRC2 inhibitors in B‐cell lymphoma treatment (Figure 7M). The BMP‐ACVR1 axis is a universal mechanism for the efficacy of PRC2 inhibitors in B‐cell lymphoma, with the example of the inhibitors used in this study, such as MAK683, EPZ6438 and HJM‐353. Loss of BMP‐ACVR1 would likely provide a resistance mechanism for PRC2 inhibitors in clinic, including tazemetostat.

PRC2 is a critical player in embryonic and organ development, regulating the expression of genes with precise spatial and temporal restrictions. Previously，studies using B cell‐specific *Ezh2^Y641F^* knock‐in mice or EZH2 silencing/inhibition in lymphoma cell lines have identified a set of PRC2 repressed genes in B‐cell lymphoma, such as p21/CDKN1A, class I and class II MHC complex components.^[^ ^16^, ^38^, ^39^ ^]^ However, there was no report on the regulation of BMP6/7 and ACVR1 by PRC2 in B cell or lymphoma. We also previously analyzed the upregulated genes by PRC2 inhibitors in lymphoma cells and did not noticed BMP or ACVR1, as there are hundreds of genes upregulated and the top genes with clear functional annotation usually catch more attention.^[^ ^23^, ^40^ ^]^ Here the integration of the transcriptomic data with CRISPR functional screen results was critical in recognizing the importance of BMP6/7 and ACVR1 upregulation. So, our data provide a strong and novel piece of evidence to comprehensively show that PRC2 represses *BMP6/7* and *ACVR1* through H3K27me3 (Figures 2 and 3). It is possible that the PRC2 suppression of BMP6/7‐ACVR1 axis is a normal function of PRC2 to maintain the GC B cells in an intricate balance of being highly proliferative but still sensitive to autocrine and external cues. The repression of class I and class II MHC complex components may be an evolutionarily conserved function of PRC2 in modulating tumor microenvironment, and the suppression of p21/CDKN1A has been explicitly shown to be required for tumorigenesis in lymphoma.^[^ ^16^, ^38^ ^]^ Interestingly, *p21/CDKN1A* is also a well‐known downstream effector of BMP signaling and pSMAD1/5 directly binds its promoter. One possibility is that pSMAD1/5 in the nucleus may better activate the expression of *p21/CDKN1A* without promotor H3K27me3 upon PRC2 inhibition, which warrants further study. In addition, the target genes of PRC2 are usually dynamic and lineage‐dependent. Recently, it was reported that EED knockout in oligodendrocyte progenitors led to the upregulation of BMP pathway in RNA‐seq data,^[^ ^41^ ^]^ aligning with the brain function of PRC2. Along these lines, further studies in GC B cell in situ are warranted.

Most B‐cell lymphomas originated from the germinal center, where mature B cells undergo SHM and rapid clonal expansion in the dark zone, and the selection with Tfh cells and follicular dendritic cells in the light zone. EZH2^Y641F^ has been reported to attenuate the interaction and dependence on Tfh cells and drive the expansion of centrocytes and the light zone size in GC.^[^ ^8^ ^]^ Interestingly, we found that primary cilia genes were upregulated by PRC2 inhibitors in tumor xenografts (Figure 6), and it has been reported that primary cilia proteins in lymphocytes are required for the dynamic structure and proper function of immune synapse.^[^ ^42^, ^43^ ^]^ Moreover, primary cilia genes were found to be repressed in GC B cells carrying *Ezh2^Y641F^* (Figure S6B–F, Supporting Information).^[^ ^8^ ^]^ It has also been shown that EZH2^Y641F^ repressed cilia genes, deconstructed cilia, and promoted metastasis in a mouse melanoma model.^[^ ^44^ ^]^ So, it is conceivable that PRC2 may similarly repress cilia‐related genes in GC B lymphocytes as in melanoma, which require cilia proteins to build an immune synapse and receive signals from Tfh cells. Validating this logic hypothesis would require delicate immune synapse detection and functional analysis in GC B cells with *Ezh2^Y641F^* or PRC2 inhibitor treatment.

As efficient tumor regression by PRC2 inhibitors requires ACVR1 (Figure 4), does BMP‐ACVR1 signaling play a role in GC B cell maturation or lymphoma repression? We checked the expression of known BMP ligands for ACVR1 and found that only BMP6 and BMP7 are expressed at high levels in lymphoma samples in CCLE and TCGA (Figure S8A, Supporting Information). BMP7 was detected in normal B cell and lymphoma, and it induced apoptosis in normal B cell.^[^ ^45^ ^]^ In a follow‐up study, GC B cell was shown to express high level of ACVR1, and BMP7 induced apoptosis and suppressed the viability‐promoting effect of CD40L but had no effect on CD40L‐induced differentiation in GC B cells.^[^ ^46^ ^]^ *ACVR1* level is lowest in mature B‐cell lymphoma among all the cancer types according to the Pan‐Cancer analysis (Figure S8B, Supporting Information).^[^ ^47^ ^]^ Inducible BMP7‐expressing lymphoma cells adopted slower DNA synthesis and proliferation, and high levels of BMP6, BMP7 or ACVR1 were associated with longer survival in DLBCL (Figure 7). Furthermore, p21/CDKN1A plays a critical role in *Ezh2^Y641F^* initiated DLBCL and is an established target gene regulated by PRC2 through H3K27me3. Meanwhile, it is also a well‐known target gene of BMP signaling.^[^ ^48^ ^]^ Therefore, a possible model incorporating all information suggests that PRC2 inhibition, on one hand, provides the building blocks for the immune synapse for proper Tfh selection and survival, and on the other hand, turns on BMP6/7‐ACVR1 signaling to induce proliferation block and apoptosis if no proper interaction occurs. The two sides may both contribute to the efficacy of PRC2 inhibitors in B‐cell lymphoma and might even strengthen each other. However, this remains a hypothesis and further studies would be required.

In summary, our results suggest that PRC2 inhibitors in B‐cell lymphoma treatment induce the upregulation of lineage differentiation, interferons and immune synapse genes, along with the activation of BMP6/7‐ACVR1 signaling, leading to proliferation block and apoptosis to render their anti‐lymphoma efficacy. BMP7 upregulation and ACVR1 signaling are not only observed in the in vitro and in vivo study results after PRC2 inhibition (Figures 2 and 3), but also correlates with better clinical survival outcomes in DLBCL patients (Figure 7). Furthermore, SMAD1 has been long recognized as a chemosensitizer in DLBCL.^[^ ^49^ ^]^ Recently, Stelling et al. reported that SMAD1 expression restoration by DNA demethylating agent decitabine would suppress DLBCL.^[^ ^50^ ^]^ Our results align well with these reports and support the critical role of BMP‐ACVR1‐pSMAD1/5 signaling in lymphoma therapy. PRC2 inhibitors show clinical efficacy in EZH2 wild‐type DLBCL patients, and the activation of BMP‐ACVR1 signaling may contribute to it. The combination of PRC2 inhibitors with R‐CHOP and other chemotherapy may also bring additional benefits to the relapsed and refractory DLBCL patients.

## 4 Experimental Section

### Cell Culture

Cells were maintained in a humidified incubator at 37 °C, 5% (vol/vol) CO2. WSU‐DLCL2, SU‐DHL‐4, Karpas422, SU‐DHL‐1, Toledo, OCI‐Ly19, and Pfeiffer cell lines were cultured in RPMI1640 (Gibco) supplemented with 15% fetal bovine serum (Lonsera # S711‐001S), 100 U mL^−1^ penicillin and 100 µg mL^−1^ streptomycin (P/S). 293T cells were cultured in DMEM (Gibco) with 10% fetal bovine serum and same concentrations of P/S.

### Cell Proliferation

WSU‐DLCL2, Karpas422, SU‐DHL‐1, Toledo, OCI‐Ly19, SU‐DHL‐4 and Pfeiffer cells were seeded in 12‐well plates at a density of 2 × 10^5^ cells mL^−1^ with the indicated concentration of MAK683, LDN‐214117 (Med Chem Express # HY‐16712) and LDN‐212854 (Med Chem Express # HY‐15897). Viable cell number was counted every 3–4 days by Vi‐CELL (Beckman Coulter). IC50 was calculated using PRISM and all proliferation experiments were repeated twice or more and the representative data were presented.

### CRISPR Screen

WSU‐DLCL2 cells stably expressing spCas9 were transduced with GeCKO v2 (A and B) human library at an MOI of 0.2 with a minimal representation of 800 transduced cells per guide RNA. One day after infection, Puromycin (4 µg mL^−1^) was included in cell culture. Cells were then split into DMSO and MAK683 (0.2 µm MAK683 dissolved in DMSO) and passaged every 2 days. Genomic DNA of cells containing 800 coverage was harvested (Qiagen # 51192) on day 14 after drug treatment. sgRNAs coding regions were recovered by PCR amplification (New England Biolabs # M0530L) in a single‐step reaction of 27 cycles. PCR products from all reactions were pooled, purified by gel extraction (Omega # D2500‐02), and sent for deep sequencing (Novogene). Total pair‐end sequences obtained on Illumina HiSeq×10 (150‐bp reads) was aligned to the human genome (hg38). Sequencing companies were commissioned to analyze the data, using MAGeCK^[^ ^51^ ^]^ (version v0.5.7) software for data comparison and difference analysis. sgRNAs MAGeCK score, sgRNAs abundance, and genes p‐value were used as the three‐way to determine reliable enrichment of genes. sgRNAs with the top 1% of MAGeCK score and the top 3000 in abundance were selected as enriched genes, which also need to have at least one enriched sgRNA and the p‐value of the gene is <0.05.

### Sequencing Data Analysis

Low‐quality sequences were removed as well as sequencing connectors in all of the samples. Sequences were aligned to the human genome (hg38). All the methods used were as previously described.^[^ ^52^ ^]^

### RNA‐Seq Data Analysis

mRNA levels of genes in triplicate samples were calculated as FPKM. Differential gene expression was determined using R package DESeq2 (version v1.20.0) with an FDR threshold of 0.05 and an Log2 Fold Change threshold of ±1.

### ChIP‐Seq DATA Analysis

Spik‐in normalization was performed on all samples. These reads were used to generate binding site with MACS2 (version 2.2.7.1) and feature counts (version v1.5.3).

### Bisulfite‐Seq Data Analysis

Sequences were aligned with Bismark (version v0.19.0), which could transform reference genome by three‐letter alignment.

### Enrichment Analysis

Fisher‐exact test was performed through Python (version 3.7.0) and its Scipy (version 1.2.1) module, the enrichment level of each gene set was calculated according to Fisher's exact test method. GSEA (Gene set enrichment analysis) is based on GSEA Desktop Application (version 4.0.3, download from http://www.gsea‐msigdb.org/gsea/downloads.jsp). All gene sets download from Molecular Signatures Database (MSigDB, http://www.gsea‐msigdb.org/gsea/msigdb/index.jsp, version v7.1/v7.2), including C2.cp/C5.go gene sets.

### Visualization

Data visualization was performed using Python's Matplotlib (version 3.1.0) and Seaborn (version 0.9.0) module, R's Gviz (version 1.30.3) package, and DeepTools software (version 3.4.3).

### Plasmids and Lentiviral Packaging

ACVR1 cDNA (BRICS#SP‐103036) was cloned into pLVX‐IRES‐ZsGreen (Clontech). ACVR1‐R206H mutation was obtained by point‐mutation PCR. shRNAs targeting the indicated genes (Table S1, Supporting Information) were cloned into the pLKO.1 (addgene#52628) vector. Lentiviruses were packaged by cotransfection of shRNA plasmids, psA2X, and pMD2G in HEK293T cells. WSU‐DLCL2 cells were infected with viral supernatants and selected with puromycin (2 µg mL^−1^) for 3 days. Knockdown efficiency was assessed by RT‐qPCR.

### Construction of the Knockout Cell Clones

Single guide RNA/DNA sequences targeting *ACVR1* (CCTCACTGAGCATCAACGATGGC), *BMPR1A* (AATCTGGATAGTATGATTCA), *BMPR2* (ATTCTTCATAGTGACACTCT), *ACVR2A* (ATGGTGACAAAGATAAACGG), and *ACVR2B* (CATCTACTACAACGCCAACT) were individually cloned into plentiCRISPRv2‐sgRNA‐Cas9 (Addgene #52961) with puromycin marker. Plasmid was packaged into lentivirus as aforementioned. WSU‐DLCL2 or SU‐DHL‐4 cells were infected by the lentivirus, selected by puromycin (2 µg mL^−1^) for 3 days, and applied to single cell sorting. The knockout clones were confirmed by RT‐qPCR and DNA sequencing.

### Quantitative PCR and ChIP‐qPCR

Total RNA was purified using TRI reagent (Sigma–Aldrich # T9424). cDNA was synthesized using M‐MLV RT kit (PROMEGA # M531A). Quantitative PCR (qPCR) was performed using ChamQ Universal SYBR qPCR Master Mix (Vazyme # Q711‐02) on a Quantstudio 7 Real Time PCR System (Applied Biosystems). The numbers of biological samples and independent experiments used for qPCR analyses are indicated in the figure legends and text. mRNA level was normalized to the expression level of GAPDH gene. Primers used for mRNA qPCR validation are listed in Table S2 (Supporting Information).

ChIP was performed with anti‐H3K27me3 (CST # 9733S) antibody following the manual of ChIP Assay Kit from Beyotime (#P2078). WSU‐DLCL2, Karpas422, SU‐DHL‐1, Toledo, and OCI‐Ly19 cells treated with or without MAK683 (5 µm) for 6 days. Formaldehyde (0.8%) was added to the culture media and incubated for 10–15 min at room temperature, rabbit IgG (Cell Signaling # 2729) was used as control. The sequences of ChIP qPCR primers are listed in Table S3 (Supporting Information).

### Western Blot

For western blot, proteins samples were extracted from cultured cells using sodium dodecylsulfate (SDS) lysate buffer (0.05 m Tris‐HCl pH 6.8, 2% SDS,10% Glycerol, 1 mm PMSF) with protease inhibitor cocktail I, phosphatase inhibitor cocktail II and III inhibitors and DTT solution. Cancer tissues were broken in Bead Mill Homogenizer and then performed as described above. Proteins were separated by SDS‐polyacrylamide gel electrophoresis (PAGE) and transferred to nitrocellulose membrane according to standard protocols. Primary and secondary antibodies were used at appropriate dilutions. Chemiluminescent detection was performed using Clarity Western ECL Substrate (Bio‐Rad‐1705061). Primary antibodies and second antibodies used were in the Table S4 (Supporting Information).

### Immunohistochemistry (IHC)

Tissues were fixed in 4% formaldehyde overnight, after dehydration embedded in paraffin. For antigen retrieval using an autoclave, the deparaffinized slides were boiled in 10 mm sodium citrate buffer (pH 6.0, Solarbio C1032‐500) maintained at 120 °C temperature for 6–8 min, according to the manufacturer's instructions. Slides were incubated overnight at 4 °C with the primary antibody Ki67 (Abcam # ab16667, 1:100) and H3K27me3 (CST # 9733S, 1:200). Slides were washed and incubated with the HRP‐conjugated goat anti‐rabbit secondary antibody (invitrogen # 31460, 1:500). The colors in all slides were developed by incubation with 3,3N‐Diaminobenzidine Tertrahydrochloride (DAB, Beyotime # P0203), following manufacturer's guidelines. After that, slides were stained with haematoxylin (Beyotime # C0107) and mounted in neutral balsam (SCR # 10004160). Images were obtained using Olympus (VS120) microscopy.

### Cell Cycle and Apoptosis Assessment

WSU‐DLCL2 and SU‐DHL‐4 cells were seeded in 12‐well plates at a density of 2 × 10^5^ cells mL^−1^ with the indicated time and concentration of MAK683, LDN‐214117 (Med Chem Express # HY‐16712) and LDN‐212854 (Med Chem Express # HY‐15897). The cells were fixed with ice cold ethanol (70%) and incubated for 2 h at 4 °C. The cells were resuspended with 50 µL RNase (0.1 mg mL^−1^) and incubated for 30 min at room temperature, then propidium iodide (0.05 mg mL^−1^) were added and incubated for 15 min at room temperature. Stained samples were analyzed by a FACS x20 sorter (BD Biosciences). The percentage of cells in each cell cycle phase and apoptosis cells was analyzed by flowjo (v10).

### Carboxyfluorescein Diacetate Succinimidyl Ester (CFSE) Staining

WSU‐DLCL2 and SU‐DHL‐4 cells were seeded in 12‐well plates at a density of 2 × 10^5^ cells mL^−1^ with the indicated time and concentration of MAK683 and LDN‐212854. According to the manufacturer's instructions, cells were resuspended with 400 µL CFSE (10 µm, Selleck # S8269) and incubated for 12–15 min at 37 °C. The fluorescent signal was detected through the GFP channel and the means of CFSE intensity were analyzed by flowjo (v10).

### EdU Incorporation Assay

WSU‐DLCL2 and SU‐DHL‐4 cells were seeded in 12‐well plates at a density of 2 × 10^5^ cells mL^−1^ with the indicated time and concentration of MAK683, LDN‐214117, and LDN‐212854. The cells were incubated with 10 µm EdU (EpiZyme # CX004L) for 8–10 h at 37 °C, following the manufacturer's instructions. The fluorescent signal was detected through the Alexa Fluor 647 channel and the percentage of EdU positive cells was analyzed by flowjo (v10).

### Mouse Xenograft Studies

All the procedures related to mouse xenograft models in this study were performed according to the guidelines and protocols approved by the Institutional Animal Care and Use Committee (IACUC) of ShanghaiTech University under the document number of 20201225001 and following the internationally recognized guidelines on animal welfare. Briefly, 1 × 10^7^ WSU‐DLCL2 cells were implanted subcutaneously in female BALB/c nude mice. Tumor length (*L*) and perpendicular width (*W*) were measured every 3–4 days with calipers and the tumor volume was calculated using the formula V = 0.5 × L × W^2^. Once the tumor volumes reached ≈200 mm^3^, mice were then block‐randomized into vehicle and treatment groups. Compound was suspended in 0.5% PHMC + 0.5% Tween 80 in water and administered orally by gavage. At the last day, mice were given the dose administration 4 h before sacrifice.

The patient‐derived xenograft studies were in collaboration with LIDE biotech and Crown Biosciences. Briefly, a surgically resected human lymphoma tissue was obtained from patient. Fresh tumor tissue fragments were collected at 4 °C in sterile HBBS supplemented with antibiotics and were cut into 1–2 mm fragments and transplanted to the BALB/c Nude mice (8 weeks old, female). Tumor volume was measured and determined similarly. Once the tumor volumes reached ≈150–200 mm^3^, mice were then block‐randomized and dosed similarly as CDX studies.

### Survival Analysis

Gene expression matrix was downloaded from TCGA (NCICCR‐DLBCL accession number phs001444). Patients’ clinical data are available in the article by R. Schmitz et al.^[^ ^36^ ^]^ All the extreme samples were removed with clinical information loss and survival of <1 year after treatment, and ultimately, clinical information was available for a total of 234 patients. Python's lifelines (version 0.21.5)^[^ ^53^ ^]^ module was used for survival analysis for all genes strongly associated with patient survival. Logrank‐test method was used to calculate p values and univariate cox regression to calculate hazard ratios (HR). The minimum P‐value^[^ ^54^ ^]^ approach was employed to find the cut point in continuous gene expression measurement for grouping patients. The data of B cell lymphoma was from PrognoScan.^[^ ^37^ ^]^

### Statistics and Reproducibility

The statistical analysis employed in each figure panels are described in the legends or in the corresponding method sections. Briefly, statistical analysis for qPCR results and IHC quantifications were carried out using GraphPad Prism 8. Data are shown as mean ± s.d.. Statistical significance was determined using the two‐tailed Student's test (t test) or ANOVA test as annotated in the legend. *p* < 0.05 was considered significant. Data distribution was assumed to be normal but was not formally tested. All cellular experiments were repeated multiple (two or more) times with biological repeats.

## Conflict of Interest

The authors declare no conflict of interest.

## Author Contributions

D.L. and Z.L. contributed equally to this work. W.Q. conceived and designed the study and most of the experiments. D.L., Z.L., and D.T. performed almost all the molecular, cellular, and animal experiments. L.C. and Y.A. performed and analyzed the ChIP‐seq and RNA‐seq experiments with the advice from L.Z., T.C., W.L., A.Z., and R. X. helped with a few cellular experiments.

## Supporting information

Supporting Information

## Data Availability

The raw data files for the sequencing analysis that generated in this study are deposited in the NCBI Gene Expression Omnibus (GEO) under the accession numbers: GSE232612, GSE232613, and GSE232614.
