# Supplementary material for: BMP‐ACVR1 Axis is Critical for Efficacy of PRC2 Inhibitors in B‐Cell Lymphoma
Source: Adv Sci (Weinh). 2024 Jan 16;11(12):2306499. doi: 10.1002/advs.202306499 (PMC10966518; doi:10.1002/advs.202306499)
Supplement: Supplementary file 1 — Supporting Information [file ADVS-11-2306499-s001.pdf]

ADVANCED SCIENCE
Open Access

Supporting Information

for *Adv. Sci.*, DOI 10.1002/advs.202306499

BMP-ACVR1 Axis is Critical for Efficacy of PRC2 Inhibitors in B-Cell Lymphoma

*Dongdong Liu, Zhen Li, Dongxia Tan, Yang An, Liping Chu, Tiancheng Chen, Weijia Li, Ailin Zhou, Ruijie Xiang, Liye Zhang, Yuxiu Qu and Wei Qi**

Supporting information

Supplementary Figures

BMP-ACVR1 Axis is Critical for Efficacy of PRC2 Inhibitors in B-Cell Lymphoma

*Dongdong Liu[1, #], Zhen Li[1, #], Dongxia Tan[1], Yang An[1], Liping Chu[1], Tiancheng Chen[1], Weijia Li[1], Ailin Zhou[1], Ruijie Xiang[1], Liye Zhang[1], Yuxiu Qu[1], Wei Qi[1, 2 *]*

[1] Gene Editing Center, School of Life Science and Technology, ShanghaiTech University, Shanghai, 201210, China

[2] Shanghai Clinical Research and Trial Center, Shanghai 201210, China

#: These authors contribute equally

*: Correspondence to: qiwei@shanghaitech.edu.cn

Supplemental Figures

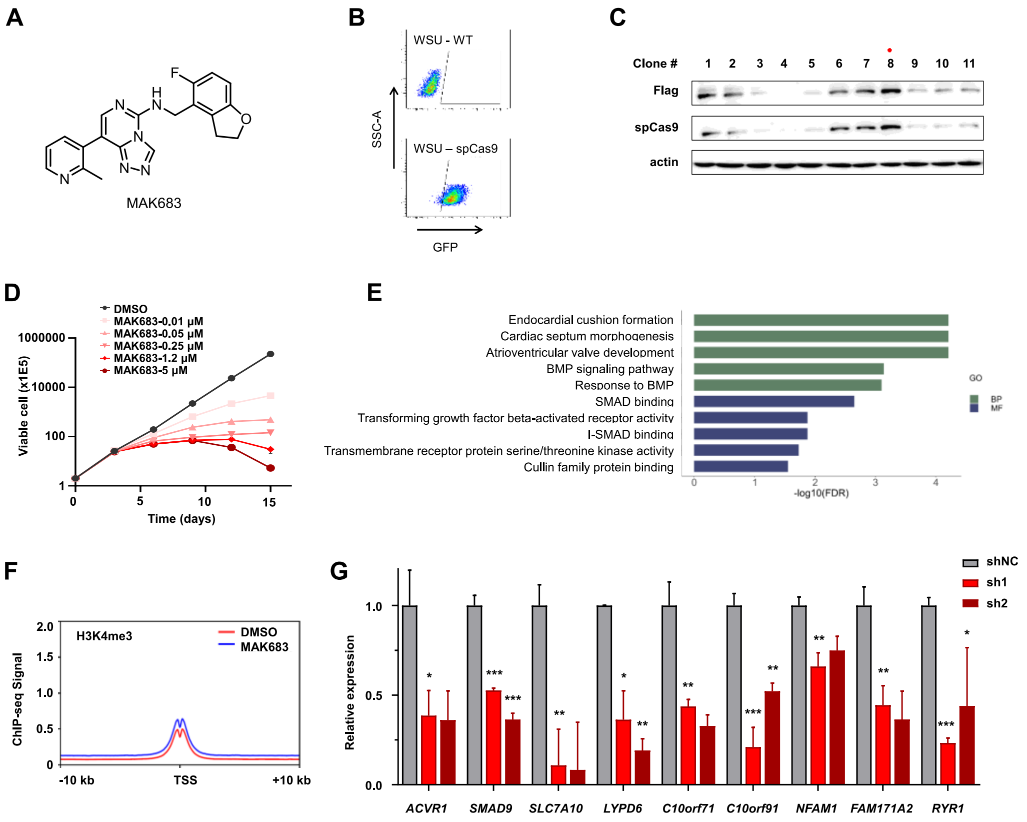

Figure S1 Combined CRISPR-Cas9 screen and transcriptome profiling reveals the critical effectors of PRC2 inhibitor in lymphoma.

(**A**) Chemical structure of MAK683. (**B**) GFP-spCas9 expression in WSU-DLCL2 was detected by FACS. (**C**) Western blots of Flag and spCas9 in GFP-spCas9 clones. Clone 8# was chosen for further experiments. (**D**) Proliferation of WSU-DLCL2 treated with MAK683 at the indicated concentrations. Viable cells were counted every 3 days (mean ± s.d., n=2). (**E**) Gene Ontology (GO) term enrichment of the 44 sgRNA targets (overlapped in **Figure 1E**). (**F**) Composite H3K4me3 profile around transcripts start sites (TSS) in WSU-DLCL2 cells treated with MAK683 at 3 μM or DMSO for 3 days. (**G**) RT-qPCR of indicated genes in WSU-DLCL2 with individual indicated shRNAs (mean ± s.d., n=2). All data were normalized to GAPDH, and shNC was arbitrarily set as 1. Statistical analysis was performed using two-tailed unpaired t test (*, $p < 0.05$; **, $p < 0.01$; ***, $p < 0.001$).

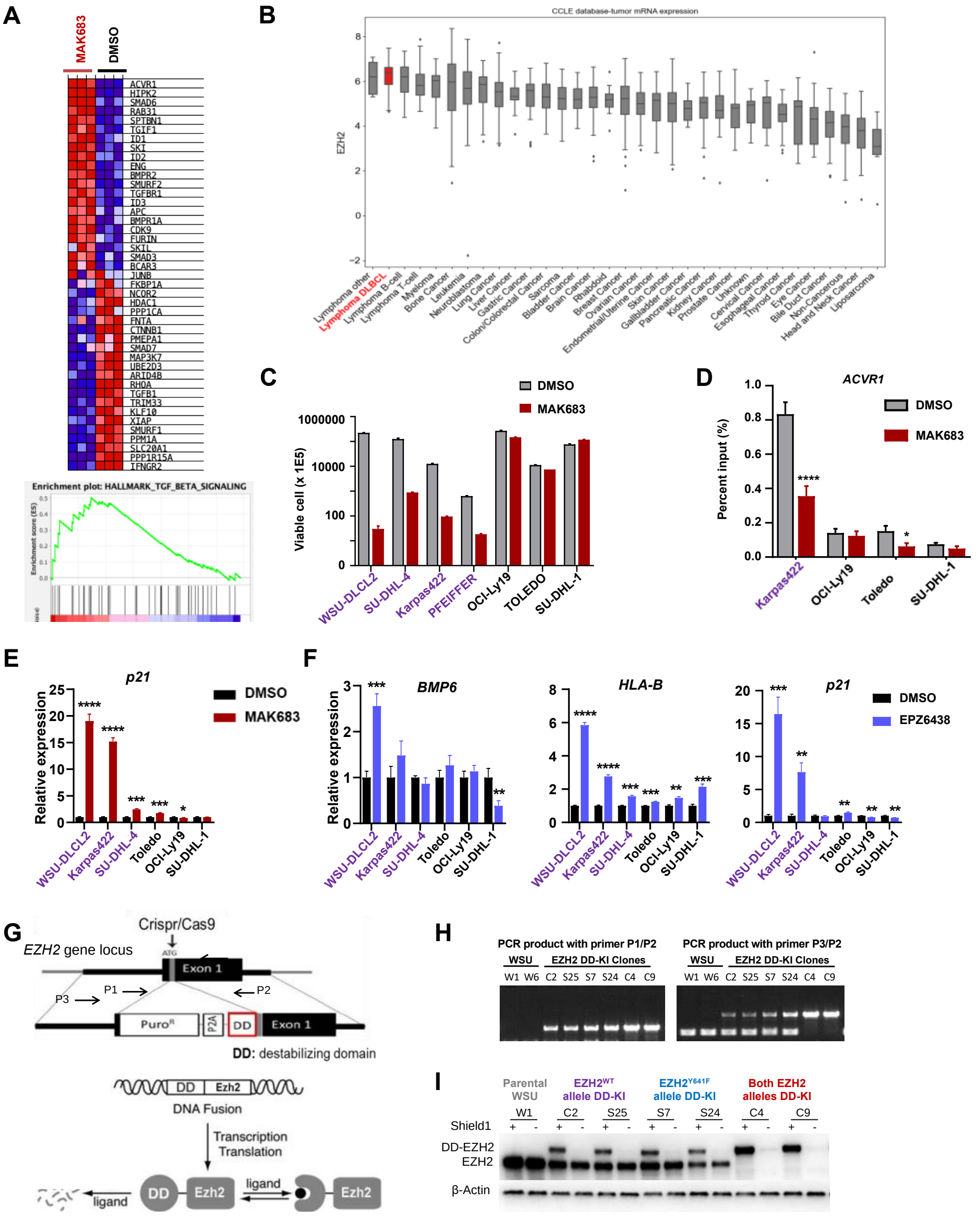

Figure S2 BMP-ACVR1 expression and signaling are under epigenetic regulation by PRC2 in lymphoma cells

(**A**) GSEA enrichment of "hallmark TGF-β signaling" and heatmap for this gene set of the RNA-seq data from WSU-DLCL2 treated with MAK683 (5 μM) or DMSO. NES: 1.406, Nominal p-value: 0.043. (**B**) *EZH2* mRNA levels in different tumor types from CCLE (red bar indicates lymphoma DLBCL). (**C**) Viable cells of WSU-DLCL2, SU-DHL-4, Karpas422, OCI-Ly19, Toledo and SU-DHL-1 treated with MAK683 (1.2 μM) or DMSO for 15 days. Pfeiffer was treated with MAK683 (with 0.01 μM) or DMSO for 12 days (mean ± s.d., n=2). (**D**) ChIP-qPCR of H3K27me3 in Karpas422, OCI-Ly19, Toledo and SU-DHL-1 cells at the *ACVR1* promoter after 6 days treatment of MAK683

(5 μM) or DMSO. Rabbit IgG was used as controls (mean ± s.d., n=3). (**E**) and (**F**) RT-qPCR of the indicated genes in WSU-DLCL2, SU-DHL-4, Karpas422, OCI-Ly19, Toledo and SU-DHL-1 cells treated with MAK683 (5 μM), EPZ6438 (5 μM) or DMSO for 4 days. All data were normalized to GAPDH, and DMSO samples were arbitrarily set as 1 (mean ± s.d., n=3). (**G**) Scheme showing the EZH2-Degron-KI strategy. (**H**) PCR analysis identified clones that have Degron-KI inserted at EZH2 locus. Location of P1/P2 and P2/P3 is illustrated in (G). (**I**) Western blots of EZH2 protein in the EZH2-DD-KI clones. All cellular experiments were performed at least two times. *P* values were determined by two-tailed unpaired t test (*, $p < 0.05$; **, $p < 0.01$; ***, $p < 0.001$; ****, < 0.0001).

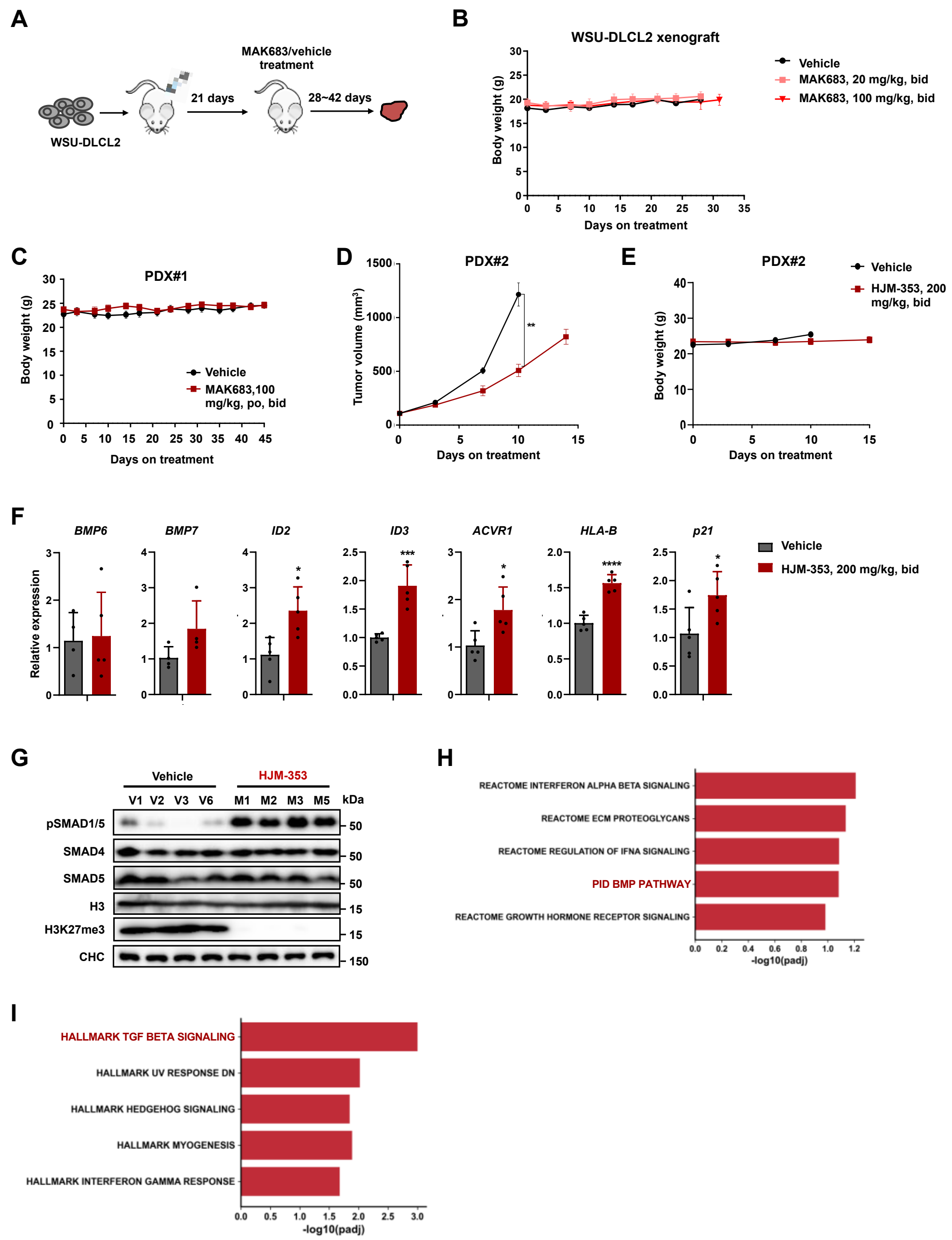

Figure S3 BMP-ACVR1 expression and signaling are upregulated by PRC2 inhibitors in lymphoma in vivo.

(**A**) Schematic view of the subcutaneous WSU-DLCL2 xenograft model. (**B**) Body weight of the mice carrying subcutaneous WSU-DLCL2 xenograft tumors in treatment of MAK683 or vehicle orally twice a day as in **Figure 3A** (mean ± s.e.m., n=5). (**C**) Body weight of the mice carrying the subcutaneous patient-derived lymphoma xenografts in treatment of MAK683 or vehicle orally twice a day as in **Figure 3G** (mean ± s.e.m., n=5). (**D**) and (**E**) Growth curve of subcutaneous DLBCL patient-derived xenograft tumors and the body weight of mice treated with HJM353 or vehicle orally twice a day (mean ± s.e.m., n=4). (**F**) RT-qPCR analysis of the

indicated genes in tumors treated as in panel (D). All data were normalized to GAPDH, and vehicle samples were arbitrarily set as 1 (mean ± s.d., n =4). **(G)** Western blots of pSMAD1/5 and the indicated proteins in PDX tumors from the end point of the study in (D). **(H)** and **(I)** Pathway enrichment analysis of RNA-seq data from the end point of the study in (D). Statistical analysis was performed using two-tailed unpaired t test (*, $p < 0.05$; **, $p < 0.01$; ***, $p < 0.001$; ****, < 0.0001).

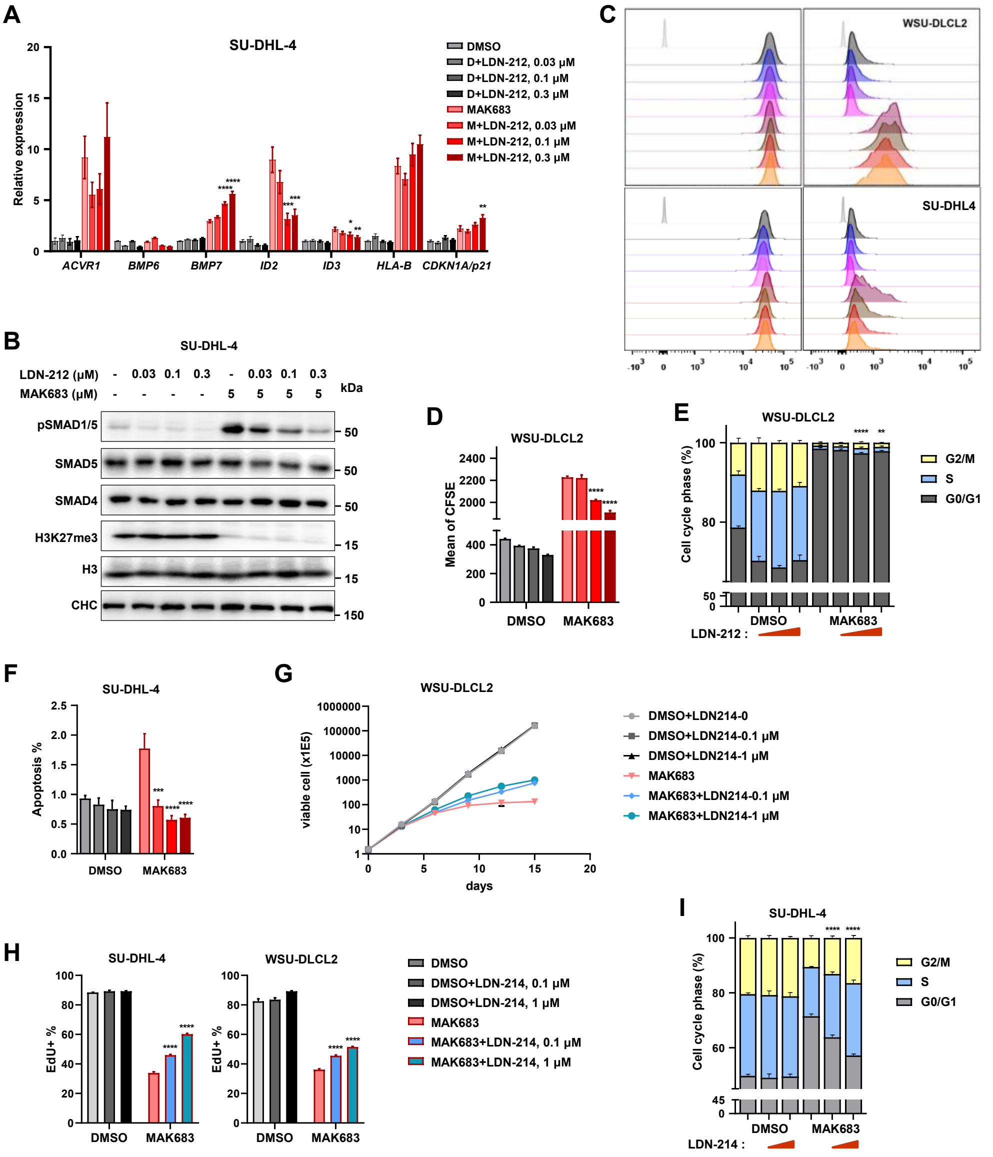

Figure S4 ACVR1 signaling promotes apoptosis and is required for the anti-lymphoma activity of PRC2 inhibitors

(**A**) and (**B**) RT-qPCR of the indicated genes and western blots of pSMAD1/5 and the indicated proteins in SU-DHL-4 cells treated with MAK683 (5 μM) and/or LDN-

212854 at the indicated concentrations. All data were normalized to GAPDH, and DMSO samples were arbitrarily set as 1 in (A**)** (mean ± s.d., n=3). (**C**) CFSE staining of the WSU-DLCL2 and SU-DHL-4 cells after incubation with LDN-212854 and/or MAK683(5 μM) for 6 days followed by detection by FACS at day 6 and day10 (n=4 and representative results are shown). (**D**) Statistic description of CFSE in WSU-DLCL2 cell as in ***c*** (mean ± s.d., n=4). (**E**) Cell cycle analysis of WSU-DLCL2 cells treated with MAK683 (5 μM) and/or LDN-212854 at the indicated concentrations for 9 days (mean ± s.d., n≥4). (**F**) Cell apoptosis in SU-DHL-4 cells treated with LDN-212854 and/or MAK683(5 μM) for 9 days, determined by sub G1-content from PI staining and FACS (mean ± s.d., n = 3). (**G**) Proliferation of WSU-DLCL2 cells treated with LDN-214117 and/or MAK683 (1.2 μM). Viable cells were counted every 3 days (mean ± s.d., n=2). (**H**) EdU incorporation analysis of WSU-DLCL2 and SU-DHL-4 cells treated with MAK683 (5 μM) and/or LDN-214117 at the indicated concentrations for 6 days respectively (mean ± s.d., n=4). (**I**) Cell cycle analysis of SU-DHL-4 cells treated with MAK683 (5 μM) and/or LDN-214117 at the indicated concentrations for 5 days (mean ± s.d., n=4). All cellular experiments were performed at least two times. Statistical analysis was performed using one-way ANOVA (*, p < 0.05; **, p < 0.01; ***, p < 0.001; ****, < 0.0001).

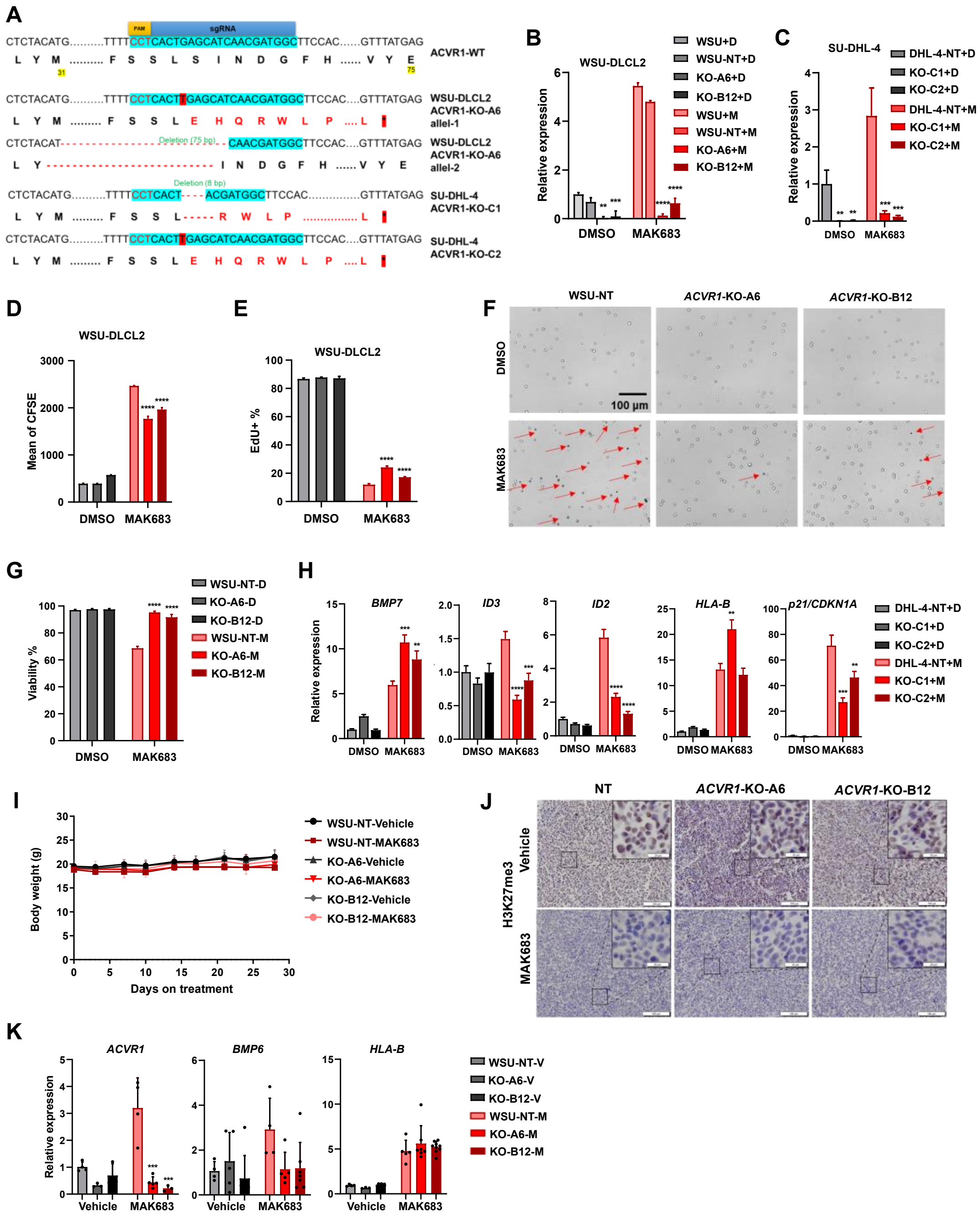

Figure S5 ACVR1 deletion compromises the anti-lymphoma activity of PRC2 inhibitors.

(**A**) DNA sequencing results of *ACVR1* knockout clones A6 (WSU-DLCL2 cells), C1 and C2 (SU-DHL-4 cells). (**B**) and (**C**) RT-qPCR analysis of *ACVR1* expression in WSU-DLCL2 clones and SU-DHL-4 clones treated with DMSO or MAK683 (1.2 μM) for 4 days. NT, a non-targeting sgRNA control clone. All data were normalized to GAPDH, and control samples were arbitrarily set as 1 (mean ± s.d., n=3). (**D**) Statistic description of CFSE in WSU-DLCL2 cells treated with MAK683 (5 μM) and/or LDN-212854 at the indicated concentrations for 10 days (mean ± s.d., n=4).

(**E**) EdU incorporation analysis of WSU-DLCL2 clones treated with MAK683 (5 μM) for 6 days (mean ± s.d., n ≥ 4). (**F**) Brightfield microscopic images of WSU-DLCL2 cells treated with DMSO or MAK683 (1.2 μM) for 15 days and stained with trypan blue. Scale bar 100 μm. Red arrow: trypan blue positive cells. (**G**) Viability percentage in WSU-DLCL2 clones (mean ± s.d., n=3). The bars are color-coded similarly in (D, E and G). (**H**) SU-DHL-4 (NT, C1 and C2) cells treated with DMSO or MAK683 (5 μM) for 4 days, RT-qPCR analysis of the indicated genes. All data were normalized to GAPDH, and NT-DMSO samples were arbitrarily set as 1 (mean ± s.d., n=3). (**I**) Body weight of the mice carrying the subcutaneous WSU-DLCL2 (NT, A6 and B12) xenografts in treatment of MAK683 or vehicle orally twice a day as in ***Figure 5I*** (mean ± s.e.m., n=5). (**J**) Representative H3K27me3 IHC images of tumor samples from the end point of study in ***Figure 5I***. Scale bar for images, 100 μm; scale bar for the intersects, 20 μm. (**K**) WSU-DLCL2 (NT, A6 and B12) cells treated with DMSO or MAK683 (5 μM) for 4 days, RT-qPCR analysis of the indicated genes. All data were normalized to GAPDH, and NT-DMSO samples were arbitrarily set as 1 (mean ± s.d., n=3). All cellular experiments were performed at least two times. Statistical analysis was performed using one-way ANOVA (*, $p < 0.05$; **, $p < 0.01$; ***, $p < 0.001$; ****, $p < 0.0001$).

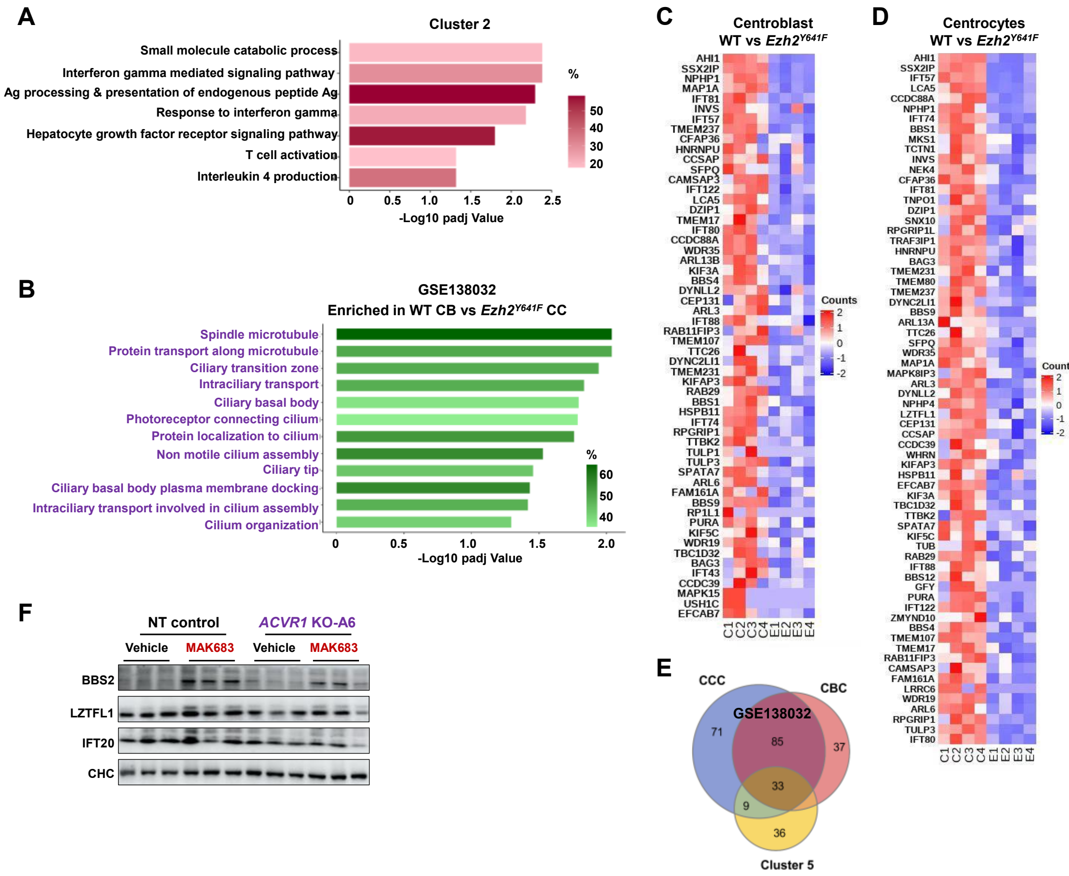

Figure S6. ACVR1 is required for MAK683-induced upregulation of immunological synapse.

(**A**) The top enriched GO pathways in the cluster 2 from the ***Figure 6F***. (**B**) GSEA analysis of upregulated genes in WT vs *Ezh2^{Y641F}* centroblasts and centrocytes (RNA-seq data from GSE138032). (**C**) and (**D**) Heatmaps depict the gene expression in WT vs *Ezh2^{Y641F}* centroblasts and centrocytes (n=4 biological replicates from GSE138032). (**E**) Venn diagram showing overlapped genes from centroblasts (GSE138032), centrocytes (GSE138032) and cluster 5 (from the ***Figure 6F***). CCC, wild type control of centrocytes, CBC, wild type control of centroblasts. (**F**) Western blots of BBS2, LZTFL1 and IFT20 in tumors samples from study in ***Figure 5I***.

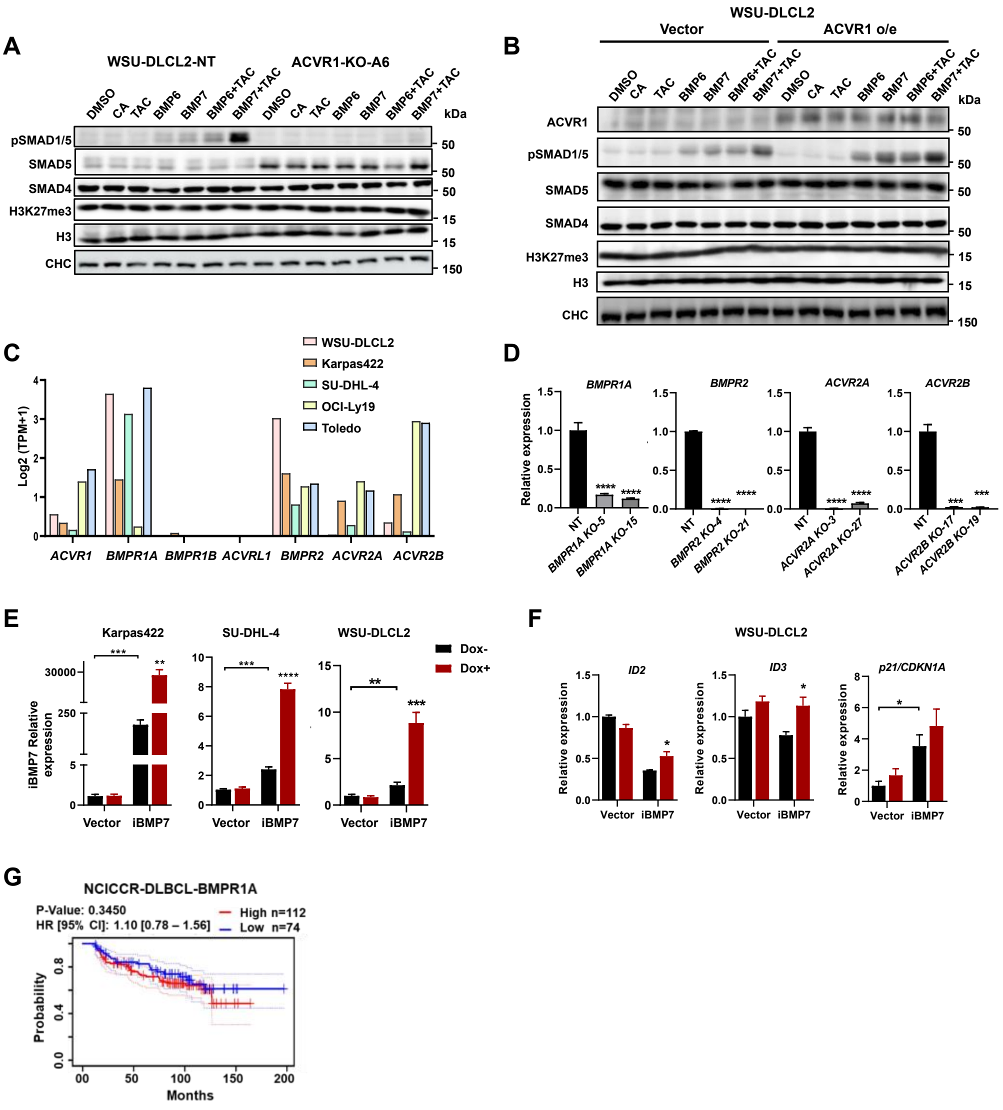

Figure S7. BMP-ACVR1 signaling is a tumor-suppressive pathway in B-cell lymphoma.

(**A**) and (**B**) Western blots of pSMAD1/5 and the indicated proteins in NT, A6 (ACVR1-KO clone), Vector, ACVR1 o/e of WSU-DLCL2 cells were treated with DMSO, Cyclosporin A (1 μg/mL), Tacrolimus (1 μg/mL), BMP6 (100 ng/mL) and BMP7 (100 ng/mL) for 45 min after starvation for 20 hours. (**C**) Expression levels of the indicated genes in WSU-DLCL2, Karpas422, SU-DHL-4, OCI-Ly19 and Toledo cells (CCLE database). (**D**) RT-qPCR of the indicated genes in the individual gene knockout clones of WSU-DLCL2. All data were normalized to GAPDH, and NT samples were arbitrarily set as 1 (mean ± s.d., n=2). (**E**) RT-qPCR of *BMP7* genes in iBMP7-WSU-DLCL2, SU-DHL-4, Karpas422 cells treated with or without doxycycline (500 ng/mL) treatment for 1 days. All data were normalized to GAPDH, and control samples were arbitrarily set as 1 in the lower panels (mean ± s.d., n ≥ 2).

(**F**) RT-qPCR of the indicated genes in iBMP7-WSU-DLCL2 cells treated as (E). All data were normalized to GAPDH, and control samples were arbitrarily set as 1 (mean ± s.d., n ≥ 2). (**G**) Kaplan-Meier survival curves of DLBCL patients stratified based on *BMPR1A* expression level (NCICCR cohort, n=112 and n=74 for *BMPR1A* hi and lo/int, respectively). All cellular experiments were performed at least two times. Statistical analysis was performed using two-tailed unpaired t test (*, $p < 0.05$; **, $p < 0.01$; ***, $p < 0.001$; ****, < 0.0001).

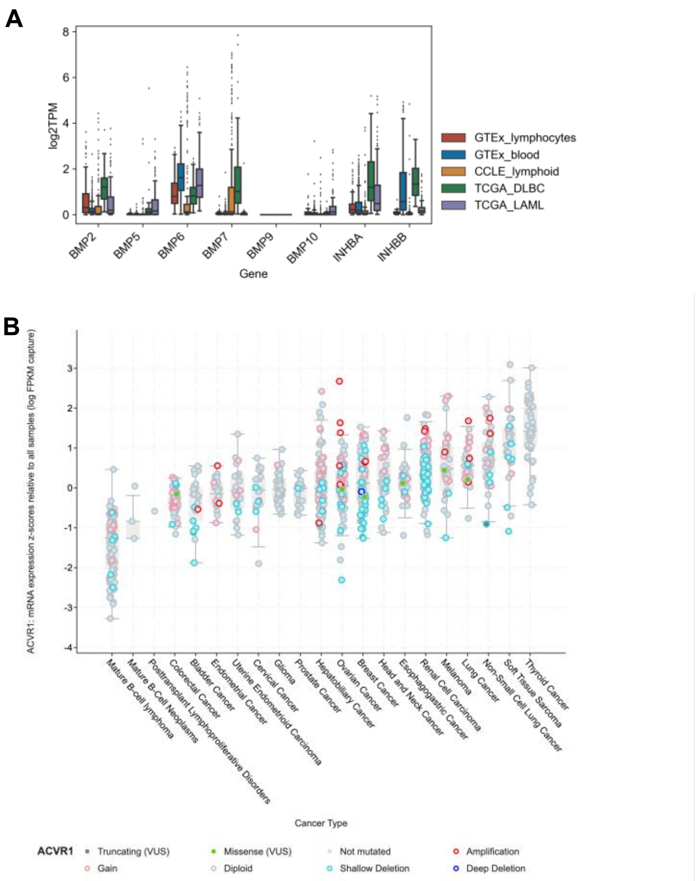

Figure S8. ACVR1-mediated signaling is a tumor suppressive pathway in B-cell lymphoma.

(A) Expression of BMP ligands in a variety of sample types (data form CCLE, GTEx and TCGA databases). (**B**) Z scores of ACVR1 expression from Pan-Cancer analysis.

Supplementary Table 1. shRNA sequences

Gene	shRNA1	shRNA2
FAM171A2	GCGCAATGGCACTGGTGTAAT	AGCATTCCAGCCTGGAGATTT
NFAM1	GTCTATGCCTGCATCGAGAAT	CCAACACAGCTATCTCCTTCA
SMAD9	GCAGAAAGAAGTGTGCATTAA	GCAACTATCAACACGGCTTCC
ACVR1	CGGATGGTGAGCAATGGTATA	GTGGATTGTTTCGATTCTTAT
C10orf71	CTAGAGGTAAGGTTGATGGAA	CAGACAGCTATCTAACTCTTA
LYPD6	CTGCTACACTCAGCACACAAT	GCCACGACGTCACCTATAAAT
SLC7A10	CAGCTTCATCTCAGAGCCTAT	GCTCATCAACTATGTGTCCTT
C10orf91	GTCTGTTAGTCAGGGTTTCAG	CAGAGAAACCTCAGACAAGAA
RYR1	GCTTCTCTAATCCGTGGCAAT	CTCTGCATCATTGGCTATAAT

Supplementary Table 2. qPCR primers

Gene	Forward Primer (5’-3’)	Reverse Primer (5’-3’)
GAPDH	AGATCATCAGCAATGCCTCCTG	ATGGCATGGACTGTGGTCATG
ACVR1	GGAAGTGGCTCTGGTCTTCC	ATACCTGCCTTTCCCGACAC
BMP6	TTCACCTTATGAACCCCGAGT	CTTCCTGGGTAATAAGGCACT
BMP7	GTGGTTGGTGGCGTTCATGTA	CAACGTGGCAGAGAACAGCAG
SMAD1	TGGTTCCAAGCAGAAGGAGG	ACAGGAGGAAGTACAGGGCT
SMAD4	CCAGCTCTGTTAGCCCCATC	TACTGGCAGGCTGACTTGTG
SMAD5	CGGCCGAGCTGCTAATAAAG	ACTTCAACAGCTTCCTTTTCGC
SMAD9	AACAACCAGCTCTTCGCTCA	TCAGCACCCCAACCCTTAAC
ID2	GACCACCCTCAACACGGATA	CACACAGTGCTTTGCTGTCA
ID3	TTCCCATCCAGACAGCCG	GCGTTCTGGAGGTGTCAGGA
P21	GAGACTCTCAGGGTCGAAAACG	TTCCTGTGGGCGGATTAGG

HLA-B	GGGATGGCGAGGACCAAAC	ACAGCTCCGATGACCACAAC
FAM171A	ATGTGTTTGGGAACCGGACT	CAGACGCATACAAGGGCAGC
NFAM1	CTAAAGAGTCGCCGCAGACA	AGATAGCTGTGTTGGCCAGG
C10orf71	TCCCCACCAAGAACACCATT	CCGTGCTGGGTTTCAGGAT
LYPD6	CGGAGCTTCACTTGCTCTTGA	GTCACTCATACAGAGGGCCTG
SLC7A10	GTGGCGCTCAAGAAGGAGAT	AGAGCAGCAGAAAGCGATGA
C10orf91	ACAGGCACCCAGGATAACTC	ACGTGCTTCTTGTCTGAGGT
RYR1	AAGCAAAATGGCTAAGGCGG	TGCAGAAATTCCCGGACCTC

Supplementary Table 3. ChIP-qPCR primers

Gene	Forward Primer (5’-3’)	Reverse Primer (5’-3’)
ACVR1	GAGCCAGTCTAGAACCCGAC	CAGTGAGGCTAGAACCTCGG
BMP6	GGGTTACATGTGGCCCTCTC	TGTAGGCGTTTAGCGGACAC
BMP7	CCGGATCAGCGATCTTCTCC	AGCTCACACGCAGTTCACTT

Supplementary Table 4. Antibody information

Name	Source	Identifier
ACVR1	Abcam	ab155981
p-SMAD1/5/9	Cell Signaling	13820S
p-SMAD1/5	Cell Signaling	9516S
SMAD1	Cell Signaling	6944S
SMAD5	Cell Signaling	12534S
SMAD4	Cell Signaling	38454S
SMAD9	invitrogen	VH3065977
H3K27me3	Cell Signaling	9733s
Histone H3	Cell Signaling	9715s
Anti-Clathrin Heavy Chain	BD Biosciences	9059557
β-Actin	Cell Signaling	3700s
ID2	invitrogen	3107BA09
Goat anti-Rabbit IgG (H+L)	invitrogen	31460
Goat anti-Mouse igG (H+L)	invitrogen	31430
